# Site and land-use associations of soil bacteria and fungi define core and indicative taxa

**DOI:** 10.1093/femsec/fiab165

**Published:** 2021-12-23

**Authors:** Florian Gschwend, Martin Hartmann, Johanna Mayerhofer, Anna-Sofia Hug, Jürg Enkerli, Andreas Gubler, Reto G Meuli, Beat Frey, Franco Widmer

**Affiliations:** Molecular Ecology, Agroscope, Reckenholzstrasse 191, CH-8046 Zürich, Switzerland; Sustainable Agroecosystems, Institute of Agricultural Sciences, Department of Environmental Systems Science, ETH Zürich, Universitätstrasse 2, CH-8092 Zürich, Switzerland; Molecular Ecology, Agroscope, Reckenholzstrasse 191, CH-8046 Zürich, Switzerland; Swiss Soil Monitoring Network NABO, Reckenholzstrasse 191, CH-8046 Zürich, Switzerland; Molecular Ecology, Agroscope, Reckenholzstrasse 191, CH-8046 Zürich, Switzerland; Swiss Soil Monitoring Network NABO, Reckenholzstrasse 191, CH-8046 Zürich, Switzerland; Swiss Soil Monitoring Network NABO, Reckenholzstrasse 191, CH-8046 Zürich, Switzerland; Rhizosphere Processes Group, Swiss Federal Institute for Forest, Snow and Landscape Research WSL, Zürcherstrasse 111, CH-8903 Birmensdorf, Switzerland; Molecular Ecology, Agroscope, Reckenholzstrasse 191, CH-8046 Zürich, Switzerland

**Keywords:** amplicon sequencing, soil microbial diversity, core taxa, core communities, environmental drivers, temporal stability

## Abstract

Soil microbial diversity has major influences on ecosystem functions and services. However, due to its complexity and uneven distribution of abundant and rare taxa, quantification of soil microbial diversity remains challenging and thereby impeding its integration into long-term monitoring programs. Using metabarcoding, we analyzed soil bacterial and fungal communities at 30 long-term soil monitoring sites from the three land-use types arable land, permanent grassland, and forest with a yearly sampling between snowmelt and first fertilization over five years. Unlike soil microbial biomass and alpha-diversity, microbial community compositions and structures were site- and land-use-specific with CAP reclassification success rates of 100%. The temporally stable site core communities included 38.5% of bacterial and 33.1% of fungal OTUs covering 95.9% and 93.2% of relative abundances. We characterized bacterial and fungal core communities and their land-use associations at the family-level. In general, fungal families revealed stronger land-use associations as compared to bacteria. This is likely due to a stronger vegetation effect on fungal core taxa, while bacterial core taxa were stronger related to soil properties. The assessment of core communities can be used to form cultivation-independent reference lists of microbial taxa, which may facilitate the development of microbial indicators for soil quality and the use of soil microbiota for long-term soil biomonitoring.

## Teaser

Soil bacterial and fungal communities at thirty sites were yearly assessed over five consecutive years and revealed temporally stable as well as land-use- and site-specific core communities.

## INTRODUCTION

Soil microorganisms constitute the majority of soil biodiversity (Bardgett and van der Putten 2014) and are main drivers of many soil processes (Costa *et al*. 2018; Hallin *et al*. 2018). A detailed understanding of belowground microbial diversity and of its influencing factors is the basis for a holistic view and understanding of ecosystem processes in terrestrial environments. However, a census of soil microorganisms remains largely incomplete, due to the enormous diversity and range of abundances of soil microorganisms. High microbial diversities have been observed at different scales ranging from aggregate (Hemkemeyer *et al*. 2018; Hemkemeyer *et al*. 2019), to landscape (Karimi *et al*. 2018), and global assessments (Bahram *et al*. 2018; Větrovský *et al*. 2019).

At the land-scape scale, soil bacterial and fungal diversities are strongly correlated to soil pH (Lauber *et al*. 2009; Griffiths *et al*. 2011), which is caused by direct effects but also by indirect effects such as changing the availability of nutrients (Glassman *et al*. 2017; Lammel *et al*. 2018). The number of bacterial taxa in soils depends on the pH and has been reported to reach its maximum at pH values between 6 and 7 (Lauber *et al*. 2009). Furthermore, community structures of soil bacteria change with pH, because specific bacterial taxa reveal distinct pH preferences. For instance, within the phylum Acidobacteria, taxa belonging to the class Acidobacteriia are in general negatively correlated to soil pH, while taxa belonging to Acidobacteria Subgroup 6 commonly reveal a positive correlation to soil pH (Kielak *et al*. 2016). Further drivers of bacterial community structures depend on the system studied and include factors such as soil texture, climate, and plant communities (Griffiths *et al*. 2016; Bahram *et al*. 2018; Karimi *et al*. 2018; Leff *et al*. 2018). In comparison to soil bacterial diversity, soil fungal diversity has been shown to be geographically more structured (Talbot *et al*. 2014; Bahram *et al*. 2018). In a global meta-analysis that covered 742 sites, Větrovský *et al*. (2019) identified climate factors as main drivers of soil fungal communities, followed by soil properties, and vegetation parameters. Finally, factors related to land management, such as agricultural intensity (Banerjee *et al*. 2019), tillage (Degrune *et al*. 2017; Babin *et al*. 2019), fertilization (Hartmann *et al*. 2015; Piazza *et al*. 2019), or compaction (Hartmann *et al*. 2014) may influence diversity of soil bacteria and fungi. While the major environmental determinants of soil bacterial and fungal communities are largely known, less is known about common components of these communities, their taxonomic representatives, and their diversities.

Surveys of soil bacterial and fungal communities usually reveal a large number of unknown taxa. Delgado-Baquerizo (2019) has reported that in a global survey 99% of bacterial and 63% of fungal OTUs remained unclassified at the species-level, and that the number of unclassified bacterial or fungal OTUs at the phylum-level in a sample has ranged between 1.4% and 9.4%. In a meta-analysis on the global diversity of soil fungi, an average of only 53% of the sequences per sample could be assigned to entries in the UNITE reference database, which notably includes sequences from environmental samples (Větrovský *et al*. 2019). High ratios of unclassified sequences at the species level may be due to a lack of resolution of the used DNA barcodes (e.g. Gschwend *et al*. 2021a), or due to missing reference sequences. To elucidate the unknown microbial diversity and describe consistently occurring OTUs, several attempts have been made to identify the most common taxa, which could constitute a core of soil microbial communities (Delgado-Baquerizo *et al*. 2018; Egidi *et al*. 2019). OTUs contributing to the global bacterial soil core community were assigned in descending order of relative abundance to the phyla Proteobacteria, Actinobacteria, Planctomycetes, Chloroflexi, Verrucomicrobia, Bacteroidetes, Gemmatimonadetes, Firmicutes, Armatimonadetes, Saccharibacteria, and candidate division WS2 (Delgado-Baquerizo *et al*. 2018). Five of these phyla, i.e. Proteobacteria, Actinobacteria, Planctomycetes, Bacteroidetes, and Firmicutes, have also been reported among those with an average relative abundance of at least 5% in a soil bacterial survey across France (Karimi *et al*. 2018), which has identified Acidobacteria as an additional dominant phylum. Dominant soil bacterial phyla have revealed distinct ecological preferences such as Alphaproteobacteria and Verrucomicrobia that were more abundant in forest and permanent grassland as compared to arable and vineyard soils, while the inverse was found for Chloroflexi and Gemmatimonadetes (Karimi *et al*. 2018). However, diverse habitat associations are often detected for taxa assigned to the same phylum. For instance within the phylum Chloroflexi, the family Anaerolineaceae were associated to soils with pH above 5, while Ktedonobacteraceae were associated to a lower soil pH (Mayerhofer *et al*. 2021). For soil fungi, a global survey of 365 sites has revealed Ascomycota, Basidiomycota, Mortierellomycota, and Mucoromycota as dominant fungal phyla in soils (Tedersoo *et al*. 2014), which has been largely confirmed, although the high abundance of Mortierellomycota has been questioned (Větrovský *et al*. 2019). Egidi *et al*. (2019) have proposed that globally dominant soil fungal OTUs almost exclusively derived from Ascomycota with 80 of 83 dominant fungal OTUs classified to this phylum. Despite the recent interest in taxonomic surveys of soil bacterial (Delgado-Baquerizo *et al*. 2018; Karimi *et al*. 2018; Walsh *et al*. 2019) and fungal diversity (Tedersoo *et al*. 2017; Egidi *et al*. 2019), habitat associations of soil bacteria and fungi at lower taxonomic levels are still largely lacking.

In a previous study, 30 long-term monitoring sites of the Swiss Soil Monitoring Network (NABO) have been surveyed over five years to define and assess long-term stability of abundant, rare, and scarce soil bacterial and fungal community components (Gschwend *et al*. 2021b). Soil bacterial and fungal communities of different sites sampled early in the vegetation period remained temporally stable and structurally distinct over five years. However, that study has not provided detailed analyses of environmental drivers of community structures among land-use types, and individual sites. Furthermore, it has focused on community structures and considered OTUs as anonymous entities without assessing their taxonomy and distributions. To develop specific microbial indicators for assessing biological soil quality, information on habitat associations of bacteria and fungi at high taxonomic resolution is needed.

Here, we assessed soil bacterial and fungal diversity at the 30 sites of the NABO with a yearly sampling between snowmelt and first fertilization over five years previously described and studied by Gschwend *et al*. (2021b). Our research goals were to (i) assess site- and land-use-specific soil microbial community measures; (ii) identify taxa, which were consistently detectable (core OTUs) and taxa, which were associated to environmental factors (indicative OTUs); (iii) assess the main environmental factors structuring core communities; (iv) describe diversity and identity of core OTUs as well as their distribution among land-use types.

## MATERIAL AND METHODS

### Sampling design, DNA extraction and microbial biomass measurement

Samples were taken during five years, from 2012 to 2016, at thirty sites (Fig. S1, Supporting Information) of the Swiss Soil Monitoring Network (NABO) in early spring after snowmelt and before fertilization. Three land-use types, i.e. arable land, permanent grassland, and forests were sampled with ten sites each. Arable sites were managed with crop rotations, which included three to six different crops, and with one exception they were conventionally tilled. Forest sites included four coniferous, two mixed, and four deciduous forests. At each site, three composite samples composed of 25 soil cores of 20 cm depth and 2.5 cm diameter were taken from a 10 m by 10 m plot according to the standardized sampling protocol of the Swiss Soil Monitoring Network (Gubler *et al*. 2019). Samples were immediately stored at 4°C after sampling and processed within 48 hours. Homogenized soil was mixed with DNA extraction buffer ([2% hexadecyl trimethyl ammonium chloride (CTAB); 20 mM EDTA pH 8; 2 M NaCl; 100 mM tris hydroxymethylaminomethane pH 8; 2% polyvinylpyrrolidone (PVP-40)], Lazzaro *et al*. 2006).

Quantitative DNA extraction was achieved by extracting DNA three times from each sample following Bürgmann *et al*. (2001) with the modifications by Hartmann *et al*. (2005). DNA quantity was determined using PicoGreen (Invitrogen, Carlsbad, CA) on a Cary Eclipse fluorescence spectrophotometer (Varian, Inc. Palo Alto, CA) and cross-validated using Qubit 1.0 (Life Technologies, Carlsbad, CA, USA). DNA was cleaned using the NucleoSpin® gDNA clean-up kit (Machery-Nagel, Düren, Germany) according to the manufacturer's instruction. Microbial biomass carbon (C_mic_) was assessed using chloroform-fumigation-extraction according to Vance *et al*. (1987) with a k_EC_ value of 0.45 (Joergensen 1996). Measurements of soil physico-chemical properties, i.e. soil pH, total and organic carbon, total nitrogen, C/N-ratio, bulk density, soil texture and gravimetric water content, have been described in Gschwend *et al*. (2021b).

### Barcode amplification, sequencing and sequence analysis

Bacterial variable region 3 and 4 of the small sub-unit of the ribosomal RNA gene (16S rRNA) were amplified using primers 341F (5′ CCTAYGGGDBGCWSCAG 3′) and 806R (5′ GGACTACNVGGGTHTCTAAT 3′) (Frey *et al*. 2016). Fungal internal transcribed spacer 2 (ITS2) was amplified using primers ITS3 (5′ CAHCGATGAAGAACGYRG 3′) and ITS4 (5′ TCCTSCGCTTATTGATATGC 3′) (Tedersoo *et al*. 2014). Four reactions using the GoTaq® Hot Start Polymerase (Promega) were done for each sample using 20 ng of DNA for each reaction. Reactions were performed according to Mayerhofer *et al*. (2017) with two modifications, which were an initial denaturation at 95°C for two minutes, as well as 35 PCR cycles for the bacterial and fungal markers. Production of sequencing libraries and paired-end sequencing on an Illumina MiSeq v3 were performed at the Génome Québéc Innovation Center at the McGill University (Montréal, Canada).

Raw sequences, (NCBI SRA: PRJNA660320) were quality filtered using a custom sequence analysis pipeline largely based on USEARCH version 9 (Edgar 2010; Frey *et al*. 2016) and is described in greater detail in Gschwend *et al*. (2021b). Only sequences occurring in at least two samples were allowed to form OTU centroids. Sequences were clustered into OTUs based on a 97% sequence identity threshold. This threshold was chosen to obtain a conservative estimate of soil microbial diversity and because diversity patterns between OTUs and sequence variants based approaches are highly correlated (Glassman and Martiny 2018). Taxonomic assignment was obtained using the RDP classifier implemented in mothur version 1.36.1 (Schloss *et al*. 2009) and a minimum bootstrap value of 80% with the SILVA 132 database (Quast *et al*. 2012) as reference for bacterial sequences. Eukaryotic sequences were classified with the same approach to a Genbank database (Frey *et al*. 2016) to discriminate between fungal and other eukaryote sequences. Fungal sequences were subsequently compared to the UNITE v 7.2 reference database (Nilsson *et al*. 2018).

### Statistics

All analyses unless stated otherwise, were performed in R (RStudio 2015; R Core Team 2016). Mean values of environmental factors were calculated for samples taken at the same time point to avoid pseudo-replication. Similarly, calculations of alpha- and beta-diversity values were based on median values of OTUs per sampling time point. Spearman correlations were used to link univariate responses to environmental factors. Multivariate responses of communities were assessed by PERMDISP (Anderson *et al*. 2006) to evaluate homogeneity of dispersions between groups and permutational analysis of variance (PERMANOVA, Anderson 2001) to analyse between group differences. PRIMER7 (Clarke and Warwick 2001; Anderson *et al*. 2008) was used for PERMANOVA. To accommodate PERMANOVA for the repeated measurements design we created a nested PERMANOVA design according to the PERMANOVA manual (Anderson *et al*. 2008) and included land-use types as a fixed factor, sites as random factor nested within land-use type, and year as a random factor. Effects on community structures were expressed as square root of component of variation (√CV), which are in the unit of the original community dissimilarity, i.e. Bray–Curtis dissimilarity. The order of covariates in sequential PERMANOVA tests were selected based on the model selection algorithm implemented in distance-based linear model (DISTLM, McArdle and Anderson 2001) within PRIMER7, where AICc was chosen as model selection criterion. *P*-values of multiple tests were adjusted using Benjamini–Hochberg procedure (Benjamini and Hochberg 1995).

Site specificity was further assessed by leave-one-out cross-validation based on linear discriminant analysis (LDA) for univariate and based on canonical analysis of principal coordinates (CAP, Anderson and Willis 2003) for community structures. LDA and CAP were calculated within R using the functions ‘train’ of the package caret (Kuhn 2008) and ‘CAPdiscrim’ of the package ‘BiodiversityR’ (Kindt and Coe 2005), respectively. For the pairwise comparisons of similarities between land-use types, median values of OTU abundances were obtained for each site followed by determining Jaccard and Bray–Curtis similarities. Median values of OTU abundances for each site were determined to avoid pseudo-replication for pairwise comparisons of land-use types. Ternary plots were drawn using the R package ggtern 3.0.0.1 (Hamilton and Ferry 2018).

### Definition of OTU groups

We distinguished two OTU groups, i.e. ‘core’ and ‘‘indicative’ OTUs, which included two or three subgroups, respectively (Table 1). Core OTUs were defined based on their consistent occurrence at a site or in a land-use type. Site core OTUs (sc-OTUs), were defined as OTUs that occur in at least 80% of the 15 samples from a given site. Similarly, land-use type core OTUs (lc-OTUs), were defined as OTUs that are sc-OTUs in at least 80% of the 10 sites of a given land-use type. Indicative OTUs included three subgroups, which were (i) correlated to an environmental factor, (ii) indicative for land-use types or (iii) indicative for an individual site. The first subgroup was defined based on a Spearman correlation of |rho| > 0.4 (*P* < 0.05) with an environmental factor. Subgroups two and three were defined based on indicator species analysis using the ‘indicspecies’ R-package (De Cáceres and Legendre 2009). OTUs with an adjusted *P*-value smaller than 0.05 and an indicator value higher than 0.8 for a single or a combination of land-use types, or for individual sites were termed ’land-use-indicative’ and ‘site-indicative’ OTUs (Table 1). Therefore, land-use- and site-indicative OTUs have a significantly higher relative abundance and occurrence in a given land-use type or site. In contrast, the definition of core OTUs does not include information of the OTU abundance and occurrence in other land-use types or sites.

**Figure 1.**
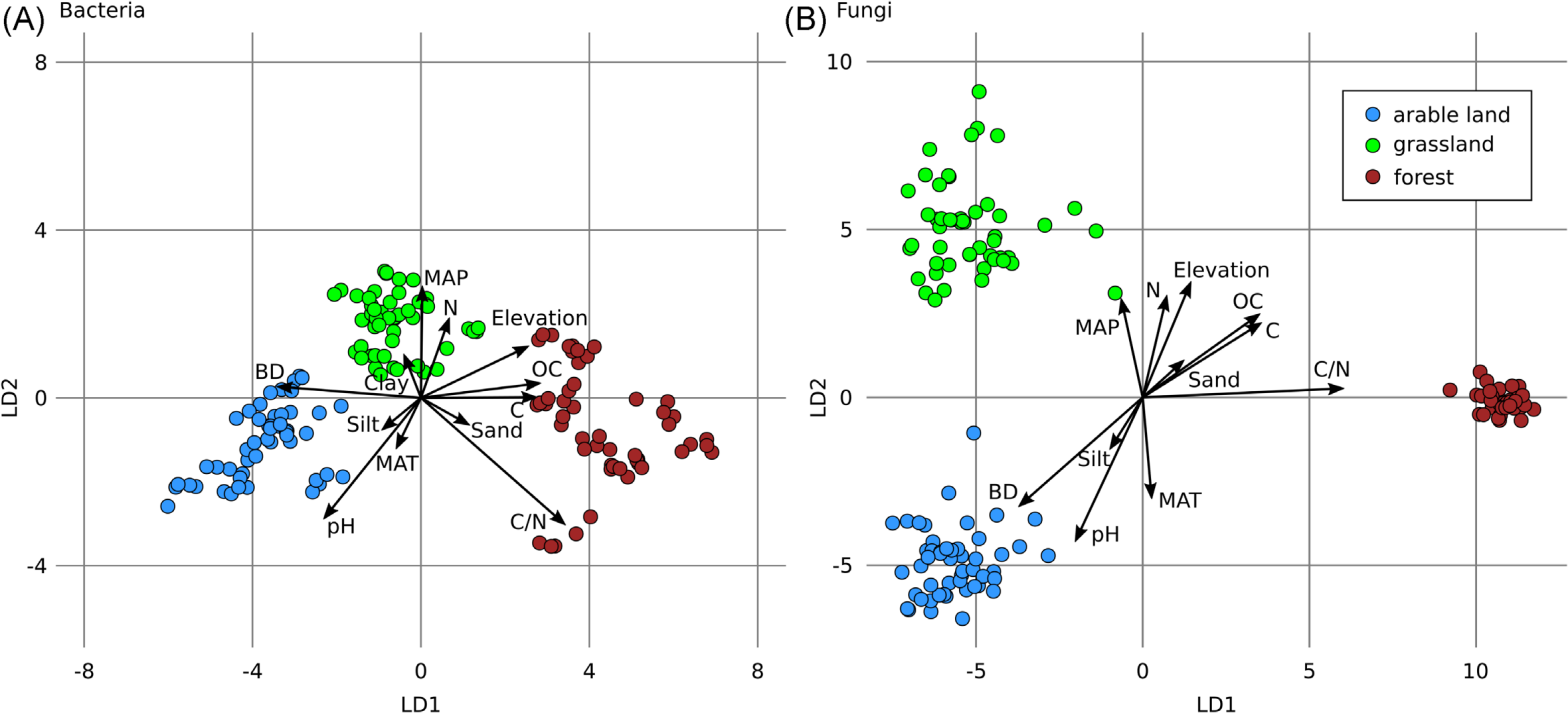
Separation of bacterial **(A)** and fungal **(B)** communities by land-use and correlated environmental factors. Three land-use types, i.e. arable land (blue), permanent grassland (green), and forest (brown), were sampled with 10 sites each. Per site, 15 samples were obtained with yearly triplicates during five years. Average communities for yearly replicates are shown (N = 150). Ordinations are based on canonical analyses of principal coordinates (CAP) constrained by land-use types. Axes show linear discriminants (LD). Arrows indicate significant correlations of communities to environmental factors, i.e. bulk density (BD), clay, silt, sand, pH, mean annual temperature (MAT), mean annual precipitation (MAP), ratio of C/N (C/N), total carbon (C) and nitrogen (N), organic carbon (OC), and elevation.

**Table 1.** Definitions of OTU groups and subgroups (see also Table 3).

OTU group	Definition
core OTUs	
site-core OTUs (sc-OTUs)	occur in at least 12 of the 15 samples from a site
land-use-core OTUs (lc-OTUs)	is a sc-OTU in at least 8 of the 10 sites of a land-use type
indicative OTUs	
environmental-factor-indicative OTUs	correlated to an environmental factor^1^ (|Spearman rho| > 0.4, *P* < 0.05)
site-indicative OTUs	indicative of an individual site (IndVal > 0.8, *P* < 0.05)
land-use-indicative OTUs	indicative of individual or combinations of land-use types (IndVal > 0.8, *P* < 0.05)

1 Environmental factors are summarized in Table S1 (Supporting Information).

## RESULTS

### Increasing resolution from microbial biomass to community structures

Thirty sites from three land-use types, i.e. 10 each from arable land, permanent grassland, and forest, were surveyed with yearly samplings during five years, which yielded 450 samples. Soil microbial communities were assessed using three different approaches, which were (i) soil microbial biomass, i.e. based on soil microbial carbon (C_mic_) content determined with chloroform fumigation extraction, and soil DNA content, that correlated (rho = 0.79, *P* < 0.0001), (ii) alpha-diversity based on OTU richness, Simpson evenness and inverse Simpson index and (iii) beta-diversity based on Jaccard similarities and Bray–Curtis dissimilarities (Table 2, see also supplementary results for a summary of the sequencing data). Microbial biomass and alpha-diversity revealed no site- (reclassification ≤ 4.7%), and low land-use-specificity (reclassification ≤ 61.3%, Table   2). Values of both microbial biomass measures were significantly reduced in arable land (Tukey HSD, *P* ≤ 0.0007; Table S1, Supporting Information), while bacterial alpha-diversity was increased in arable land (Tukey HSD, *P*  =  0.0096; Table S1, Supporting Information). Fungal alpha-diversity with the exception of fungal OTU richness were significantly lower in forest soils (Tukey HSD, *P* ≤ 0.01; Table S1, Supporting Information). Community compositions (Jaccard similarity) and structures (Bray–Curtis dissimilarity) were land-use- (Fig. 1) and site-specific with reclassification success rates of 100% for bacteria and fungi (Table 2). To resolve different drivers of soil bacteria and fungi, information on community compositions or structures was needed rather than bulk parameters such as microbial biomass or alpha-diversity.

**Table 2.** Site and land-use specific soil microbial communities at different analytical levels. Site and land-use type specificity was calculated using a leave-one-out reclassification test based on linear discriminant analysis for univariate and canonical analysis of principal coordinates (CAP) with 9999 permutations for community compositions and structures.

		LUT^1^		Site	
Community parameter	Taxon	Reclass^2^	*P*-value	Reclass^2^	*P*-value
Organic Carbon		60.7%	6.95 * 10^–12^	4.7%	0.235
Microbial biomass					
C_mic_^3^		60.0%	2.13 * 10^–11^	4.0%	0.384
DNA		61.3%	2.20 * 10^–12^	2.0%	0.880
Alpha diversity					
OTU richness	Bacteria	50.7%	8.82 * 10^–6^	0.7%	0.994
Simpson evenness	Bacteria	54.0%	1.57 * 10^–7^	4.0%	0.384
Inverse Simpson	Bacteria	57.3%	1.45 * 10^–9^	0.7%	0.994
OTU richness	Fungi	28.0%	0.931	4.7%	0.235
Simpson evenness	Fungi	42.0%	0.016	0.0%	1.000
Inverse Simpson	Fungi	40.7%	0.036	0.0%	1.000
Beta diversity					
Jaccard similarity	Bacteria	100%	0.0001	100%	0.0001
Bray–Curtis dissimilarity	Bacteria	100%	0.0001	100%	0.0001
Jaccard similarity	Fungi	100%	0.0001	100%	0.0001
Bray–Curtis dissimilarity	Fungi	100%	0.0001	100%	0.0001

1 LUT = Land-use type

2 Reclass.: Reclassification success of leave-one-out tests.

3 C_mic_: carbon content based on chloroform fumigation extraction.

### Partitioning of OTUs into core and indicative groups

The high site-specificity of soil bacterial and fungal community structures, which was maintained over five years, also reflected a high temporal stability. Temporally stable core taxa, i.e. site-core (sc) OTUs and land-use-core (lc) OTUs were defined as outlined in Table 1. Of the 18 140 bacterial OTUs (bOTUs) 6979 (38.5%), which covered 95.9% relative abundance were classified as sc-OTUs and 1136 of these sc-OTUs (covering 69.1% relative abundance) were also classified as lc-OTUs (Table 3). A similar proportion of the 8477 fungal OTUs (fOTUs), i.e. 2802 fOTUs (33.1%) and covering 93.2% relative abundance, was classified as sc-OTUs, but only 103 of them (29.4% relative abundance) were also classified as lc-OTUs. In addition to these core taxa, we defined indicative OTUs, i.e. OTUs that structured communities according to environmental conditions. More specifically, we distinguished three categories of indicative OTUs, i.e. OTUs correlated to environmental factor, as well as OTUs indicative for land-use types and OTUs indicative of a given site (see Table 1 for definitions). Most strikingly, the number and particularly the abundance of site-indicative OTUs was higher for fungi (1445 fOTUs, 29.9% relative abundance), as compared to bacteria (1146 bOTUs, 3.1% relative abundance). The vast majority of indicative OTUs were also classified as sc-OTUs (95% for bacteria, 90% for fungi, Fig. S2, Supporting Information). Communities composed of only sc-OTUs, i.e. core communities, were almost perfectly correlated (rho ≥ 0.97) to the entire communities, both in terms of alpha- and beta-diversity (Table S2, Supporting Information). Consequently, soil microbial core communities are representative of the respective entire communities. The following analyses were therefore based on these core communities.

**Table 3.** Summary of OTU partitioning into core and indicative groups and subgroups. Core and indicative OTUs were defined at the site and the land-use type level (see Table 1 for definitions of OTU groups and subgroups). LUT = land-use type.

	OTU group	Subgroup	OTUs [N]	Abundance [%]	Correlation^1^ [rho]	Phyla [N]	Families [N]
Bacteria	Core	Site	6 979	95.9	1.000	31	215
	Core	LUT	1 136	69.1	0.995	17	119
	indicative	Environmental factor	3 103	67.0	0.983	27	164
	indicative	Site	1 146	3.1	0.736	28	106
	indicative	LUT	699	27.2	0.931	17	102
	All		18 140	100		46	320
Fungi	Core	Site	2 802	93.2	0.999	9	176
	Core	LUT	103	29.4	0.893	5	35
	indicative	Environmental factor	553	42.5	0.942	7	96
	indicative	Site	1 445	29.9	0.765	7	125
	indicative	LUT	171	35.2	0.891	5	50
	All		8 477	100		12	304

1 Spearman correlation to entire community (Mantel test).

### Environmental factors driving structures of core communities

Soil habitats of different land-use types were characterized by distinct environmental factors. Arable sites were characterized by increased soil pH, and bulk density, and forest sites were characterized by increased carbon contents and C/N-ratios (Fig. 1), while grassland sites generally revealed intermediate levels of the assessed environmental factors (Table S1, Supporting Information). Soil bacterial and fungal core communities were mainly structured by soil pH and the C/N-ratio (Table 4). In addition to the environmental factors considered, land-use type and site significantly explained variance of soil bacterial (√CV_Land-use type_ = 0.23, √CV_Site_ = 0.31), and fungal (√CV_Land-use type_ = 0.31, √CV_Site_ = 0.49) community structures. Soil pH was the strongest driver for bacterial community structures overall and within each land-use type (Table S3, Supporting Information). The second strongest environmental factor in the overall analysis was the C/N-ratio, but it had no or minimal effects on the community structures within land-use types (Tables S3 and S4, Supporting Information). This may be due to the clear difference in C/N-ratio between forest and the other two land-use types (Table S1, Supporting Information), indicating that a high C/N-ratio represented a proxy for forest soils in the overall analysis. The separate analysis of arable sites also allowed to consider crop as an additional factor shaping microbial communities (Tables S3 and S4, Supporting Information), which was more strongly affecting fungal (√CV = 0.16) as compared to bacterial (√CV  =  0.06) core communities. In line with the data on core community structures, the strongest correlations of individual OTUs to environmental factors were detected with soil pH for bacterial OTUs (Table S5, Supporting Information) and with soil pH, C/N-ratio, and organic carbon for fungal OTUs (Table S6, Supporting Information).

**Table 4.** Effects of environmental factors on bacterial (A) and fungal (B) communities as assessed by PERMANOVA. Factors are sorted by their position in the PERMANOVA model with environmental factors as covariates. Year and site were random factors with site being nested within land-use type. Factors below the line are categorical. Significance codes: *** *P* < 0.001, ** *P* < 0.01, * *P* < 0.05

(A) Bacteria					(B) Fungi				
Env. factor^1^	Pseudo-F	√CV ^2^	*P*-value		Env. factor^1^	Pseudo-F	√CV^2^	*P*-value	
pH	18.6	0.25	0.0001	***	C/N-ratio	5.5	0.19	0.0001	***
C/N-ratio	5.4	0.15	0.0001	***	pH	2.6	0.14	0.0001	***
MAP^3^	2.2	0.07	0.0001	***	Elevation	1.5	0.09	0.0086	**
Clay	2.0	0.06	0.0001	***	Sand	1.3	0.06	0.0330	*
Elevation	1.4	0.05	0.0119	*	Clay	1.2	0.06	0.1399	
C_org_	1.5	0.07	0.0130	*	MAT^4^	1.2	0.06	0.1012	
Sand	1.3	0.06	0.0338	*	C_org_	1.2	0.06	0.1226	
MAT^4^	1.2	0.04	0.1418		Bulk density	1.3	0.08	0.1061	
Year	4.2	0.05	0.0001	***	Year	2.2	0.06	0.0001	***
LUT^5^	2.9	0.23	0.0017	**	LUT^5^	2.5	0.31	0.0001	***
Site	19.4	0.31	0.0001	***	Site	14.4	0.49	0.0001	***
LUT^5^ x Year	1.5	0.04	0.0001	***	LUT^5^ x Year	1.3	0.05	0.0001	***
Residuals		0.15			Residuals		0.28		

1 Env. factor: environmental factor;

2 √CV: square root of component of variation, expressed as Bray–Curtis dissimilarity;

3 MAP: mean annual precipitation

4 MAT: mean annual temperature

5 LUT: Land-use type

### Association of bacterial and fungal core OTUs to land-use types

**Figure 2.**
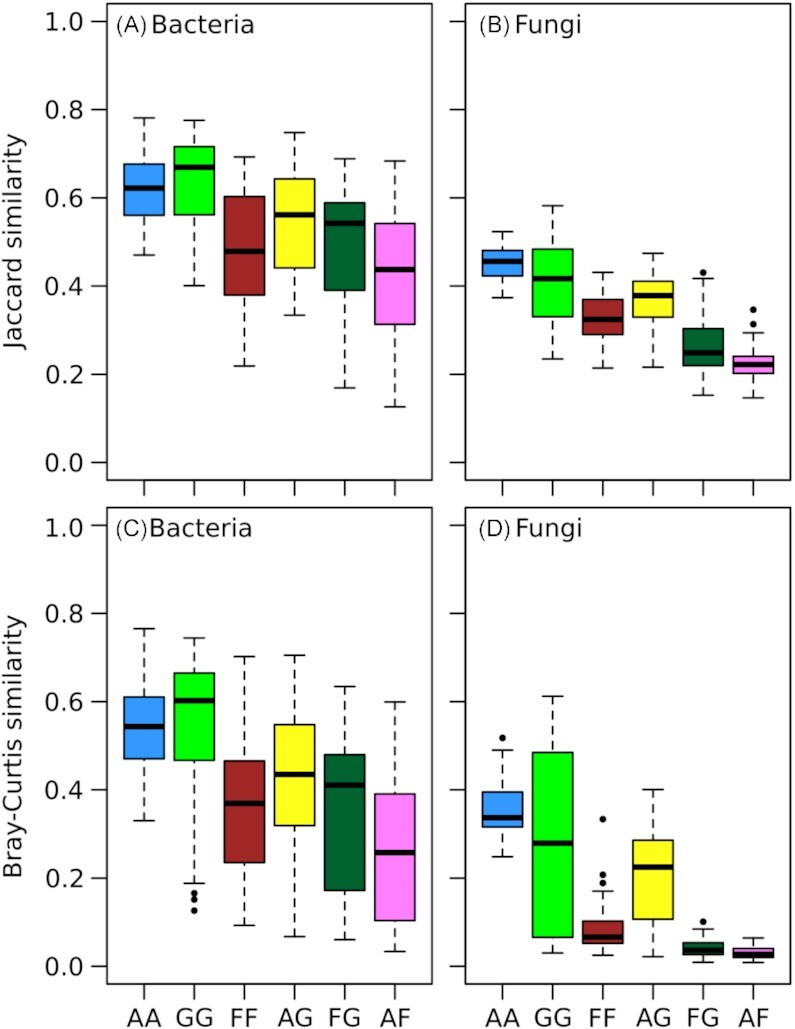
Pairwise comparisons of bacterial **(A, C)** and fungal **(B, D)** communities composed of core OTUs for a site, i.e. OTUs that occurred in at least 12 of the 15 samples from a site. Boxplots showing Jaccard (A, B) and Bray–Curtis (C, D) similarities between two sites depending on their land-use type. The Jaccard similarity corresponds to the ratio of shared OTUs between two sites, while the Bray–Curtis similarity takes also the relative abundance of each OTU into account. Sites of three land-use types, i.e. arable land (A), grassland (G), and forest (F), were assessed in pairwise combinations of the same land-use type (AA, GG and FF) as well as between different land-use types (AG, FG and AF).

**Figure 3.**
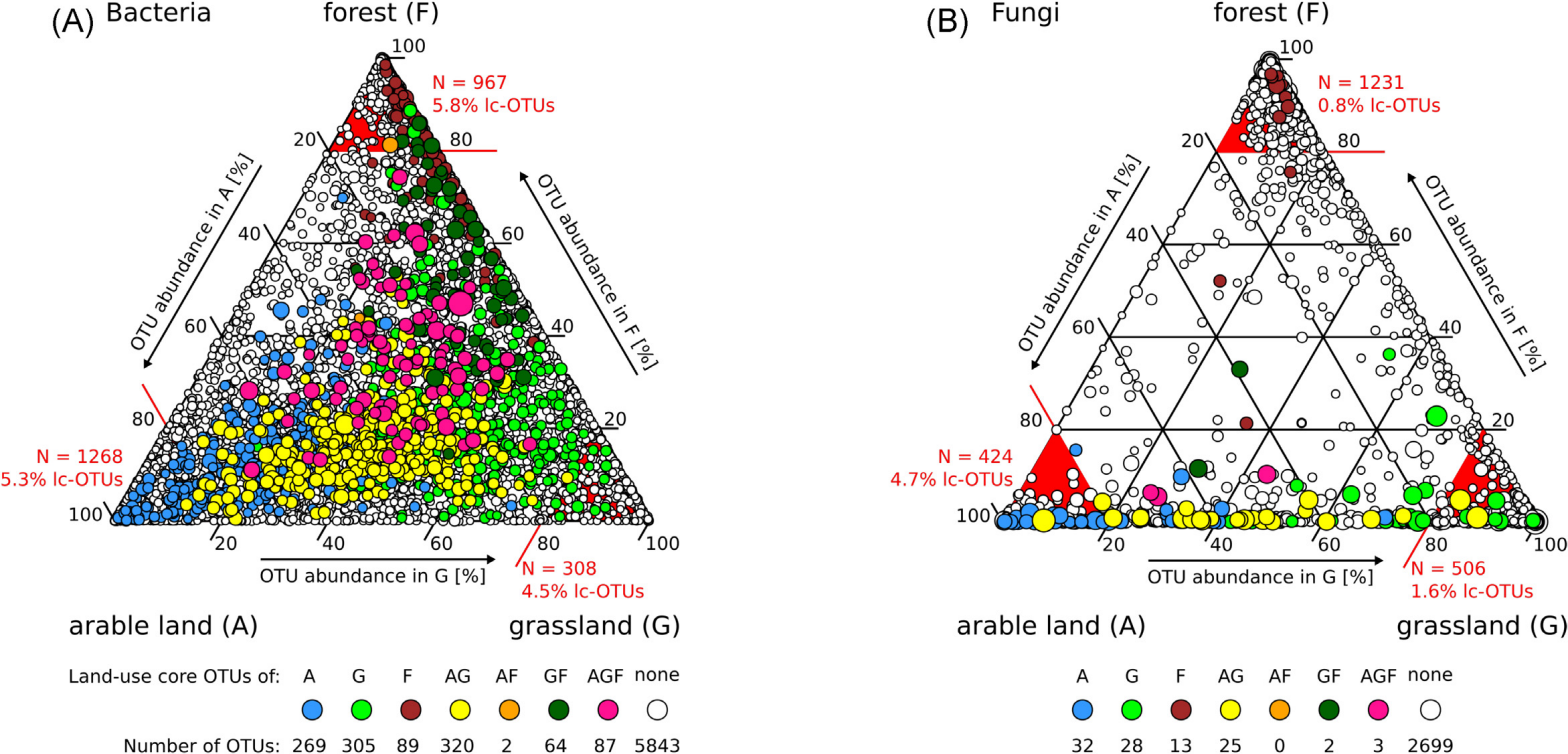
Ternary plots showing occurrences of the 6979 bacterial **(A)** and 2802 fungal **(B)** site core OTUs in the three land-use types and their combinations. Circles represent site core OTUs (sc-OTUs) and circle sizes indicate their relative abundance. Colored OTUs represent sc-OTUs, which are also land-use-core OTUs (lc-OTUs) from individual or combination of land-use types. White circles correspond to sc-OTUs, which are not part of land-use-core communities. The numbers of lc-OTUs of each land-use type or land-use type combinations are indicated below the ternary plots. The sc-OTUs were defined as OTUs occurring in 12 of 15 samples from a site and lc-OTUs as OTUs, which are sc-OTUs in 8 of 10 sites from a land-use type. Red lines and red triangles highlight the plot area, in which sc-OTUs occur which obtain at least 80% of their sequences from the respective single land-use type. The number of these sc-OTUs and the % of lc-OTUs among these are indicated in red at the corners of the ternary plots.

The similarities of bacterial and fungal communities among land-use types were highest between arable and permanent grassland soils, while they were lowest between arable and forest soils (AG and AF in Fig. 2). The similarity between communities from forest and permanent grassland sites was higher for bacteria than for fungi, which was particularly striking, when relative abundances were considered as accounted for in Bray Curtis similarities (FG in Fig. 2C and D). To assess these differences in greater detail, the distribution of core taxa among the land-use types were analyzed using ternary plots, which depict the abundance of sc-OTUs in each land-use type and in all combinations (Fig. 3). The ternary plots clearly revealed different distributions of bacterial and fungal sc-OTUs among land-use types. On the one hand, bacterial sc-OTUs were distributed among the land-use types and all their combinations except for the combination of ‘arable land and forest’, for which only two lc-OTUs were detected (Fig. 3A). Eighty-seven bacterial sc-OTUs were core of all three land-use types (AGF in Fig. 3A). On the other hand, fungal sc-OTUs were accumulated along the axes of arable land to permanent grassland and in forest (Fig. 3B). Only three fungal sc-OTUs were cores of all three land-use types and no land-use type core was detected for the combination of arable land and forest (Fig. 3B). The difference in bacterial and fungal distributions among the land-use types was also evident from the number of sc-OTUs with at least 80% of their abundance in a single land-use type (Fig. 3, red tips of the ternary plots). For bacteria, the number of such sc-OTUs that not necessarily represented an lc-OTU, was highest in arable soils (1239 bOTUs), slightly less in forest (967 bOTUs), and lowest in permanent grassland (308 bOTUs). For fungi, more sc-OTUs were predominantly detected in forest (1231 fOTUs), as compared to permanent grassland (502 fOTUs) and arable land (424 fOTUs).

### Distribution of bacterial and fungal families among land-use types

**Figure 4.**
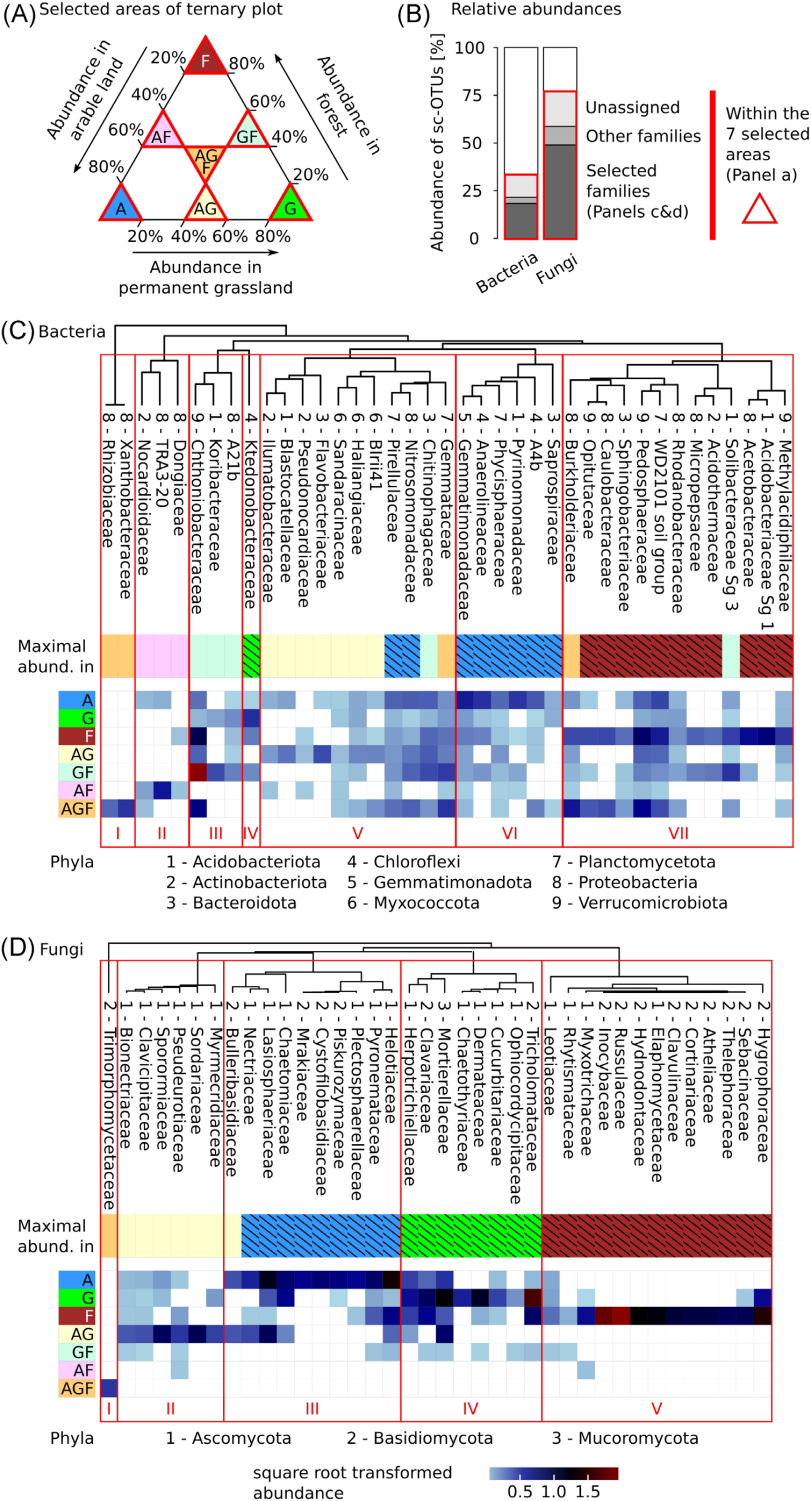
Distribution of most abundant bacterial and fungal families among land-use types. Based on the ternary plots (Fig. 3) site-core OTUs (sc-OTUs) were selected from seven areas **(A)** corresponding to sc-OTUs with at least 80% of their abundance in a single land-use type (A, G, F), with at least 40% in each of two land-use type (AG, GF, AF), or with at least 20% in each land-use types (AGF). The proportions of relative abundances covered by the selected sc-OTUs, and their assignment at the family level, is shown in panel **(B)**. Panels **(C)** and **(D)**show the relative abundances of the ten most abundant bacterial (C) and fungal (D) families of each area of the ternary plots. Light blue indicates low, dark blue middle, and brown high relative abundances. White areas represent absences of families in an area of the ternary plot. The area in which a family has its highest abundance is indicated by the following color code (Maximal abund.): blue (A), green (G), brown (F), yellow (AG), light green (GF), pink (AF), and orange (AGF). Highest abundances in a single land-use type are indicated by black hatching. Dendrograms show clustering of normalized relative abundances of families in the land-use types and their combinations using average clustering (UPGMA). Red boxes highlight clusters of families with similar distributions among land-use types.

For taxonomic characterization of core communities, we focused on the family level, since the classified OTUs can be more reliably assigned at this level and since the number of unclassified OTUs increased at lower taxonomic levels. For instance, 50.7% of the bacterial and 47.1% of the fungal OTUs were unclassified at the family-level, while these numbers were 78.0% for bacterial and 60.3% for fungal OTUs at the genus-level. In order to analyze associations of families to land-use types, we extracted sc-OTUs that were predominantly associated to a single or combinations of land-use types based on the ternary plot (Fig. 4A). The ten most abundant families in each of the seven areas specified in the ternary plot, i.e. triangles A, G, F, AG, GF, AF and AGF, were extracted. They covered in the selected areas 18.7% and 49.2% of the overall relative abundance of bacterial and fungal sc-OTUs, respectively, (Fig. 4B, dark grey area) and resulted in a list of 39 bacterial and 38 fungal families (Fig. 4C and D). Cluster analysis was used to group these families according to their distribution patterns in the land-use types, which yielded seven bacterial and five fungal clusters (Fig. 4C and D). All clusters composed of at least three families included families of several phyla with the exception of the fungal cluster II that was exclusively composed of families from the Ascomycota, but which were not closely related to each other (Fig. S3, Supporting Information). Furthermore, all phyla represented by at least three families were detected in at least two clusters with the exception of Myxococcota (Fig. 4). All three families from the Myxococcota, i.e. BIrIi41, Haliangiaceae and Sandaracinaceae, were included in bacterial cluster V and occurred in all three land-use types, but showed a preference for ‘arable land and grassland’ (Fig. 4C). More homogenous representations of land-use types within the clusters were found for fungi as compared to bacteria. Most strikingly, fungal cluster V, which was composed of families such as Myxotrichaceae, Inocybaceae, and Russulaceae, occurred most strongly and almost exclusively in forest soils. Clusters predominantly associated to permanent grassland included only one bacterial family, the Ktedonobacteraceae (cluster IV, Fig. 4C), but eight fungal families, e.g. Mortierellaceae and Chaetothyriaceae (cluster IV, Fig. 4D). Within the clusters, also groupings with more resolved land-use type associations were revealed. For instance, within fungal cluster IV the fungal families Mortierellaceae, Clavariaceae and Herpotrichiellaceae were all most abundant in permanent grassland but revealed a complex occurrence pattern in many land-use types, while the fungal family Chaetothyriaceae was exclusively detected in permanent grassland soils. Similarly, within fungal cluster III, which was mainly associated to arable land, some families such as Lasiosphaeriaceae and Nectriaceae were also prominently detected in the combination ‘arable land and permanent grassland’ while the Bulleribasidiaceae, as an exemption in cluster III, were more abundant in the combination ‘arable land and permanent grassland’ but comparably abundant in ‘arable land’. For bacteria, such clear clustering was less pronounced. Cluster VI exclusively associated to ‘arable land’ but for instance in cluster VII only 11 of the 13 families were most abundant in forest soils. Within cluster VII, families such as Pedosphaeraceae or the candidate WD2101 soil group were also commonly detected in arable and permanent grassland soils. The strongest forest associations were observed for families Acidobacteriaceae Subgroup 1 as well as Acetobacteraceae, Methylacidiphilaceae, Acidothermaceae and Micropepsaceae. Therefore, stronger associations to land-use types or their combinations were detected for fungi as compared to bacteria. This was further supported by the number of families with their highest abundance in a single land-use type (A, G or F), which was lower for bacteria (20, Fig.   4C) as compared to fungi (30, Fig. 4D).

To detect families, which showed the strongest and most consistent associations to land-use types, we compared core and indicative OTUs. More specifically, we first selected OTUs, which were core and indicative of the same land-use type or land-use type combinations and aggregated these OTUs at the family-level. This yielded 304 bacterial (Table S7, Supporting Information) and 58 fungal OTUs (Table S8, Supporting Information). Then, we selected families, which included at least four (Bacteria) or two (Fungi) OTUs that were both core and indicative of the same land-uses (Table 5). This resulted in 16 bacterial and 9 fungal families (Table 5), which were also among the families described in Fig. 4, with the exception of bacterial candidate groups SC-I-84 and AKYH767, as well as the fungal family Phaeosphaeriaceae. Two bacterial families, Anaerolineaceae and Pyrinomonadaceae included arable core and indicative OTUs and a single bacterial family, Acidobacteriaceae Subgroup 1, included only forest core and indicative OTUs. No bacterial family included only OTUs that were core and indicative of permanent grassland soils. Among fungi Chaetomiaceae and Myxotrichaceae included only OTUs that were core and indicative of a single land-use type, i.e. arable land and forest, respectively. No fungal family included exclusively OTUs that were core and indicative of permanent grassland soils. Furthermore, no bacterial and fungal OTUs were core and indicative of the combination ‘arable land and forest’ and only bacterial but no fungal families included OTUs that were core and indicative of ‘permanent grassland and forest’. The lack of such OTUs is consistent with the few sc-OTUs detected in the corresponding areas of the ternary plots (Fig. 3), as well as with low similarities of bacterial and fungal communities among arable and forest sites, and equally low similarities among fungal communities of permanent grassland and forest sites (Fig. 2).

**Table 5.** Number of OTUs, which were indicative (IndVal >0.8,  *P* < 0.05) and core for the same land-use types from selected bacterial and fungal families. Families were selected if at least four (bacteria) or two (fungi) OTUs were indicative and core for the same land-use type or land-use type combination. All families are shown in Tables S7 (Bacteria) and S8 (Fungi), Supporting Information. Associations of families to land-use types are indicated according to  Fig. 4. Stars indicate families, which have the highest number of indicative and lc-OTUs and the highest abundance in the same land-use type or land-use type combination.

	Indicative and lc-OTUs^1^ (all indicative OTUs)						Fig. 4	
Family	A	AG	G	AF	GF	F	Cluster	Main abund.^2^
Bacteria								
Chthoniobacteraceae	1(1)	4(12)	0(1)	0(0)	2(5)	3(4)	III	GF
Pirellulaceae	4(5)	1(8)	0(0)	0(0)	0(2)	0(0)	V	A*
Chitinophagaceae	4(10)	3(12)	2(2)	0(0)	1(4)	1(3)	V	GF
Gemmatimonadaceae	5(9)	3(5)	0(0)	0(0)	0(2)	1(1)	VI	A*
Anaerolineaceae	4(4)	0(3)	0(0)	0(0)	0(1)	0(0)	VI	A*
Pyrinomonadaceae	4(4)	0(0)	0(0)	0(0)	0(1)	0(0)	VI	A*
Burkholderiaceae	1(2)	4(7)	0(0)	0(0)	0(1)	3(4)	VII	AGF
Pedosphaeraceae	4(5)	7(11)	3(3)	0(0)	1(12)	3(5)	VII	F
WD2101 soil group	2(5)	9(18)	1(2)	0(0)	1(4)	1(5)	VII	F
Acidobacteriaceae Sg 1	0(0)	0(0)	0(0)	0(0)	0(1)	6(8)	VII	F*
Acetobacteraceae	0(0)	0(0)	0(0)	0(0)	2(2)	4(6)	VII	F*
Caulobacteraceae	0(0)	1(1)	0(0)	0(0)	0(0)	3(5)	VII	F*
Acidothermaceae	0(0)	0(0)	0(0)	0(0)	2(3)	2(4)	VII	F*
Solibacteraceae Sg 3	0(0)	0(2)	0(0)	0(0)	3(12)	1(3)	VII	GF*
AKYH767	0(0)	4(4)	0(0)	0(0)	0(0)	0(0)		
SC-I-84	1(1)	2(4)	2(2)	0(0)	0(3)	0(0)		
Others (incl. unclassified)	47(75)	53(195)	12(21)	0(4)	28(110)	17(41)		
All	86(128)	105(287)	23(29)	0(4)	42(162)	48(89)		
Fungi								
Pseudeurotiaceae	0(0)	1(1)	0(0)	0(0)	0(0)	1(1)	II	AG*
Lasiosphaeriaceae	3(4)	2(7)	0(1)	0(0)	0(0)	0(0)	III	A*
Plectosphaerellaceae	2(3)	1(1)	0(0)	0(0)	0(0)	0(0)	III	A*
Chaetomiaceae	2(2)	0(0)	0(1)	0(0)	0(0)	0(0)	III	A*
Nectriaceae	1(1)	3(9)	0(0)	0(0)	0(1)	0(0)	III	A
Helotiaceae	0(2)	3(4)	0(0)	0(0)	0(0)	0(1)	III	A
Mortierellaceae	0(0)	2(5)	1(1)	0(0)	0(0)	1(1)	IV	G
Myxotrichaceae	0(0)	0(0)	0(0)	0(0)	0(0)	2(4)	V	F*
Phaeosphaeriaceae	1(1)	1(1)	0(0)	0(0)	0(0)	0(0)		
Others (incl. unclassified)	7(35)	12(53)	5(13)	0(2)	0(0)	7(20)		
All	16(47)	25(79)	6(16)	0(2)	0(1)	11(26)		

1 indicative and lc-OTUs: OTUs, which are indicative and core of the same land-use type(s), note that OTUs cannot be indicative of all sites, i.e. the combination AGF;

2 Main abund.: Main abundance in arable land (A), permanent grassland (G), forest (F) or their combinations, according to Fig. 4.

## DISCUSSION

### Land-use-specificity of soil bacterial and fungal communities

Soil bacterial and fungal communities were surveyed during 5 years at 30 sites of the Swiss Soil Monitoring Network including three different land-use types, i.e. arable land, permanent grassland, and forest. This revealed communities that were highly specific to land-use types and sites, and which were stable over five years. A detailed analysis on the long-term stability of these communities early in the vegetation period without considering intra-annual variability has already been described (Gschwend *et al*. 2021b). Here, we focused on the environmental drivers that shape this land-use- and site-specificity of soil bacterial and fungal communities, as well as on their taxonomic compositions.

Each land-use type was characterized by differences in the combinations of soil properties, management, and vegetation (Table S1, Supporting Information). In arable soils, pH and bulk density were increased, while carbon contents were equal or lower than in permanent grassland and forest soils. Furthermore, management of arable soils included crop rotations, tillage (except one site), mineral and organic fertilization, as well as plant protection, which are known to influence soil bacterial and fungal communities (Hartmann *et al*. 2015; Rivera-Becerril *et al*. 2017; Peralta *et al*. 2018). Microbial biomass was significantly reduced in arable soils as compared to permanent grassland and forest soils (Table S1, Supporting Information), which confirms earlier findings (Dequiedt *et al*. 2011). Bacterial communities in arable soils were characterized by families such as Anaerolineaceae, Pyrinomonadaceae and Gemmatimonadaceae. Anaerolineaceae are widely distributed in soils, and particularly prevalent in low-oxygen environments, e.g. in compacted soils (Hartmann *et al*. 2014) or paddy fields (Jiao *et al*. 2019). As they may act as indicators for soil oxygen depletion (Gschwend *et al*. 2020) and as they have been recently detected to be associated with soil compaction in arable fields (Longepierre *et al*. 2021), their high abundance in arable soils may be a sign of soil compaction in arable land due to common management practices with heavy machinery. The families Haliangiaceae, Sandaracinaceae and BIrii41 revealed similar distributions among land-use types with the highest abundance in arable and grassland soils (Fig. 4C). This is in agreement with findings by Karimi *et al*. (2018), who detected the genus *Haliangium* in all samples and who found its highest relative abundance in arable and grassland soils. All three families are classified within the candidate phylum Myxococcota, which regroups many predatory bacterial species (Waite *et al*. 2020), and represented the only phylum in which all selected families revealed similar habitat preferences in our survey (Fig. S3a, Supporting Information). Fungal communities in arable soils were for instance characterized by Lasiosphaeriaceae, Plectosphaerellaceae, Chaetomiaceae and Mrakiaceae. With the exception of the basidiomycetous yeasts Mrakiaceae and Cystofilobasidiaceae (Liu *et al*. 2015), fungal families associated to arable soils also occurred in permanent grassland soils (Fig. 4). For instance, Plectosphaerellaceae that include important soil-borne plant pathogens such as *Verticillium* (Giraldo and Crous 2019) had two lc-OTUs that were also indicative for arable land, as well as one that was indicative for ‘arable land and permanent grassland’ (Table 5). In these cases, OTUs assigned to the same family have distinct land-use type associations, which may for instance be driven by species-specific host plant preferences (Klosterman *et al*. 2009).

Permanent grassland soils were characterized by soil property values, which lay between those of arable and forest soils (Table S1, Supporting Information). Their management included fertilization, mowing, and grazing, which may change soil bacterial and fungal community structures (Kaiser *et al*. 2016; Cui *et al*. 2020; Gilmullina *et al*. 2020). A single bacterial family, the Ktedonobacteraceae (phylum Chloroflexi) had their highest abundance in the permanent grassland section of the ternary plot, but also occurred in forest and less in arable soils (Fig. 4C). Ktedonobacteraceae are aerobic, mycelium-forming bacteria and contain a single genus with one described species, i.e. *Ktedonobacter racemifer*, which was isolated from soil of a black locust forest in Italy (Cavaletti *et al*. 2006). Metabarcoding of bacterial communities from 2173 soil samples across France revealed sequences assigned to *Ktedonobacter* in 80% of all samples (Karimi *et al*. 2018). Families that characterized fungal communities in permanent grasslands included for instance the grassland-specific Chaetothyriaceae (Fig. 4D). Chaetothyriaceae include mainly epiphytic species living on plants (Quan *et al*. 2020) suggesting that their distribution may depend on host plants. However, in a survey of switchgrass-associated fungal communities, OTUs attributed to this family have also been detected associated to the switchgrass roots and adjacent soils, but not on plant leaves (Lee and Hawkes 2020), indicating that Chaetothyriaceae also include soil fungi.

Forest soils were characterized by relatively high contents of carbon, higher C/N-ratios, and lower soil pH as compared to the arable soils (Table S1, Supporting Information). Bacterial families associated to forest soils included Acidobacteriaceae Subgroup 1, Acetobacteraceae, Acidothermaceae, as well as the more widely distributed WD2101 soil group, and Pedosphaeraceae (Fig. 4C, Table 5). Acidobacteriaceae Subgroup 1 have been repeatedly reported to negatively correlate with soil pH (Kielak *et al*. 2016) and revealed increased abundances in soils with a pH below 6.5 (Jones *et al*. 2009). Acetobacteraceae have also been reported to strongly and negatively correlate with soil pH and to have higher abundances in forest as compared to grassland soils (Nacke *et al*. 2011). Therefore, soil pH, which is well known to be a major driver of soil bacterial communities (e.g. Lauber *et al*. 2009; Karimi *et al*. 2018), was the main factor determining forest associated soil bacterial taxa. Fungal communities in forest soils were mainly composed of ectomycorrhizal families such as Russulaceae, Inocybaceae, and Clavulinaceae, which is in agreement with previous findings (e.g. Frey *et al*. 2021). Thirteen fungal families were strongly associated to forest (Cluster V, Fig. 4D), but only one of these, the Myxotrichaceae, included indicative OTUs of forest soils (Table S8, Supporting Information). This is likely explained by the different forest ecosystems including deciduous, mixed and coniferous forests that have been sampled. As ectomycorrhizal fungi depend on their host tree species (Bahnmann *et al*. 2018), none of these families occurred at eight or more forest sites and were thus not generally indicative for forest soils. Myxotrichaceae included for instance *Oidiodendron* spp., which were repeatedly detected among the abundant soil fungi in metabarcoding surveys of Swiss forest soils (Hartmann *et al*. 2017; Frey *et al*. 2020), and which are common saprobes in acid soils but some of which also form ericoid mycorrhiza (Rice and Currah 2005). Therefore, their widespread and indicative distribution in various forest soil ecosystem may relate to a dependence on understory vegetation, or on the general preference for acidic soils.

### Similarities of soil bacterial and fungal communities among land-use types

The similarities among soil bacterial communities from different land-use types were lowest for the combination of arable land and forest (Fig. 2), which was also the only land-use type combination for which no bacterial lc-OTU was indicative (Table 5). Similarities between soil bacterial communities from arable and permanent grassland soils corresponded to values observed between permanent grassland and forest soils (Fig. 2). This suggests that soil bacterial communities represented a sequential order following the soil properties and the land-use intensity from arable land, to permanent grassland and forest. For fungi, similarities from communities of permanent grassland and forest soils were equally low as among communities of arable and forest soils (Fig. 2). Furthermore, no fungal OTUs was found that was indicative and land-use core for the combination ‘permanent grassland and forest’ or the combination ‘arable land and forest’ (Table 5). Therefore, soil fungal, unlike bacterial, communities revealed little overlap (Bray–Curtis < 0.10, Fig. 2) between permanent grassland and forest soils. Considering dissimilarities among communities as proxies for the transfer of soil microorganisms among sites allows describing the structure of their metacommunities (Beck *et al*. 2019; Wisnoski and Lennon 2021). In this view, soil bacterial communities of arable, permanent grassland, and forest soils formed a single metacommunity, which was characterized by a continuous change from arable land, to permanent grassland and forest. Soil fungal communities, however, formed two metacommunities, one created by fungal communities of arable and permanent grassland soils and the other by fungal communities of forest soils.

The distinct structures of soil bacterial and fungal metacommunities can be explained by different factors influencing their community assembly. On the one hand, bacterial communities were more strongly structured by soil properties and climatic factors as compared to soil fungal communities (Table 4). On the other hand, soil fungal communities were more strongly structured by vegetation as compared to soil bacterial communities. For instance, acidophilic bacterial families predominantly occurred in forest soils (Fig. 4C), while ectomycorrhizal fungal families dominated soil fungal communities in forest soils (Fig. 4D). Confirming our results Frey *et al*. (2021) reported stronger effects of tree species on fungal as compared to bacterial community structures. Stronger vegetation effects on soil fungal as compared to bacterial communities were also revealed in the other land-use types, as crops had a stronger effect on soil fungal as compared to bacterial community structures (Tables S3 and S4, Supporting Information), which is in agreement with the findings of Ai *et al*. (2018). Stronger legacy effects of different grassland mixtures on soil fungal as compared to soil bacterial communities have been described in a grassland field experiment (Fox *et al*. 2020), which further supports the stronger impact of plants on soil fungal as compared to bacterial communities.

### Potential use of sc-OTUs to provide a temporally stable, cultivation-independent reference list of dominant taxa

Site core OTUs accounted for 38.5% of bacterial and 33.1% of fungal OTUs, but covered 95.9% and 93.2% of relative abundance (Table 3). As sc-OTUs occurred in at least four of the five years, the large majority of retrieved sequences, could be attributed to temporally stable OTUs. These sc-OTUs not only were temporally stable but also included 95% of bacterial and 90% of fungal indicative OTUs (Fig. S2, Supporting Information) and were representative of the diversities of entire communities (Table S2, Supporting Information). Furthermore, OTUs largely restricted to a single site were classified as site-indicative OTUs, which was the case of 14.5% of bacterial and 44.8% of fungal sc-OTUs. Therefore, the majority of sc-OTUs were consistently detectable at several sites revealing their potential to act as reference set for the analysis of soil microbial diversity in soil habitats similar to those assessed in this study. Such reference sets are of particular interest for predictive modelling of soil bacterial and fungal diversity and distribution (Jiao *et al*. 2019), and may also be used as reference values for long-term soil quality monitoring (Gschwend *et al*. 2021b), although their intra-annual variability remains to be assessed. Furthermore, they may constitute a set of taxa that can be screened to find robust bioindicators for specific soil functions, such as plant pathogen suppression (Trivedi *et al*. 2017). Currently, long-term monitoring systems of soil biodiversity are largely lacking (Leeuwen *et al*. 2017; Guerra *et al*. 2020), which is particularly concerning given the ongoing environmental changes and the central role of soil biodiversity for global ecosystem processes. Finally, sc-OTUs provide support to establish lists of the most characteristic soil microorganisms, for which cultivation strategies or whole-genome sequencing are particularly valuable (Carini 2019). Currently, still too few dominating soil bacterial and fungal taxa have cultured representatives or available genome sequences, which would enable more detailed insight into their functions in the ecosystem (Delgado-Baquerizo *et al*. 2018; Egidi *et al*. 2019; Steen *et al*. 2019).

## CONCLUSIONS

While microbial biomass and alpha-diversity measures at thirty long-term monitoring sites revealed only few differences among land-use types and sites, community compositions (Jaccard similarity) and structures (Bray–Curtis dissimilarity) yielded characteristic descriptors for each land-use type and site. Therefore, resolution obtained by metabarcoding were necessary to accurately describe soil bacterial and fungal communities. Temporally stable core OTUs accounted for 95.9% of bacterial and 93.2% of fungal sequences. These core OTUs were representative of entire communities and showed responses to distinct habitats. In total 4184 indicative bacterial and 1968 indicative fungal OTUs, of which 95% and 90% were also temporally stable core OTUs, were identified. These yield promising targets for the development of microbial indicators for robust soil quality analyses. Bacterial and fungal families were identified that revealed strong associations to one or more land-use types. In general, fungal families revealed stronger associations to land-use types, which may be explained by the stronger influences of vegetation on fungi as compared to bacteria, whereas bacteria were more strongly correlated with soil properties. Consequently, metacommunities of soil bacteria and fungi were differently structured. On the one hand, bacterial communities represented a sequential order following soil properties and land-use intensity from arable land, to permanent grassland and forest. On the other hand, fungal communities of forest sites showed only minor similarities to those from arable land and permanent grassland sites. The robustly assessed and temporally stable core OTUs may serve as references for future surveys of soil bacterial and fungal diversity. This may facilitate long-term soil quality monitoring by detecting disturbances of the characteristic habitat associated core communities, and it may also enable the development of predictive modelling for metabarcoding based soil quality analyses.

## Supplementary Material

fiab165_Supplemental_Files
