# Supplementary material for: Site and land-use associations of soil bacteria and fungi define core and indicative taxa
Source: FEMS Microbiol Ecol. 2021 Dec 23;97(12):fiab165. doi: 10.1093/femsec/fiab165 (PMC8752248; doi:10.1093/femsec/fiab165)
Supplement: fiab165_Supplemental_Files [file fiab165_supplemental_files.docx]

# Supplements

1. Supplementary Results
2. Supplementary Figures and Tables

# Supplementary results: Sequence data overview

In total, 9 020 192 bacterial and 11 958 695 fungal high-quality sequences were obtained with at least 11 791 bacterial and 7 719 fungal sequences per sample and on average 20 045 (standard deviation: ± 3 705) bacterial and 26 575 (± 8 780) fungal sequences per sample. The average Good’s coverages were 0.92 (± 0.022) for bacteria and 0.98 (± 0.005) for fungi. Sequences were grouped into 18 140 bacterial OTUs (bOTU) and 8 477 fungal OTUs (fOTU) with an average of 2 714 (± 658) bOTUs and 562 (± 134) fOTUs per sample. Bacterial OTUs were assigned to 46 phyla, of which 31 occurred in core communities (Table S9) and fungal OTUs to 12 phyla of which 9 occurred in core communities (Table S10).

# Supplementary Figures and Tables

Supplementary figures


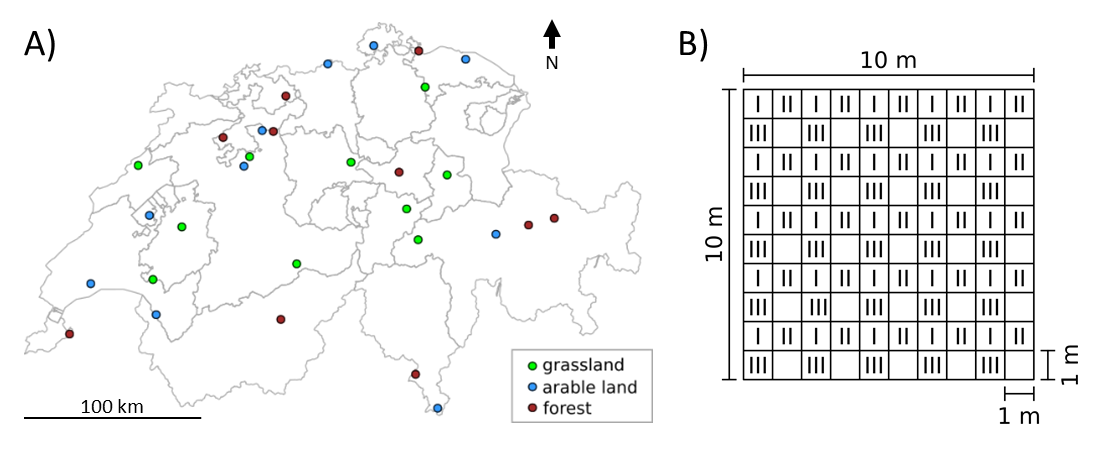


Figure S1: Map of Switzerland showing the thirty sampling sites and their land-use type (A). At each site three composite samples (I – III) were taken within a 10 m x 10 m area (B). Each composite sample was composed of 25 cores of 2.5 cm diameter and 20 cm depth. In each square meter marked with I to III, one core was randomly taken and mixed with other cores of the same number.


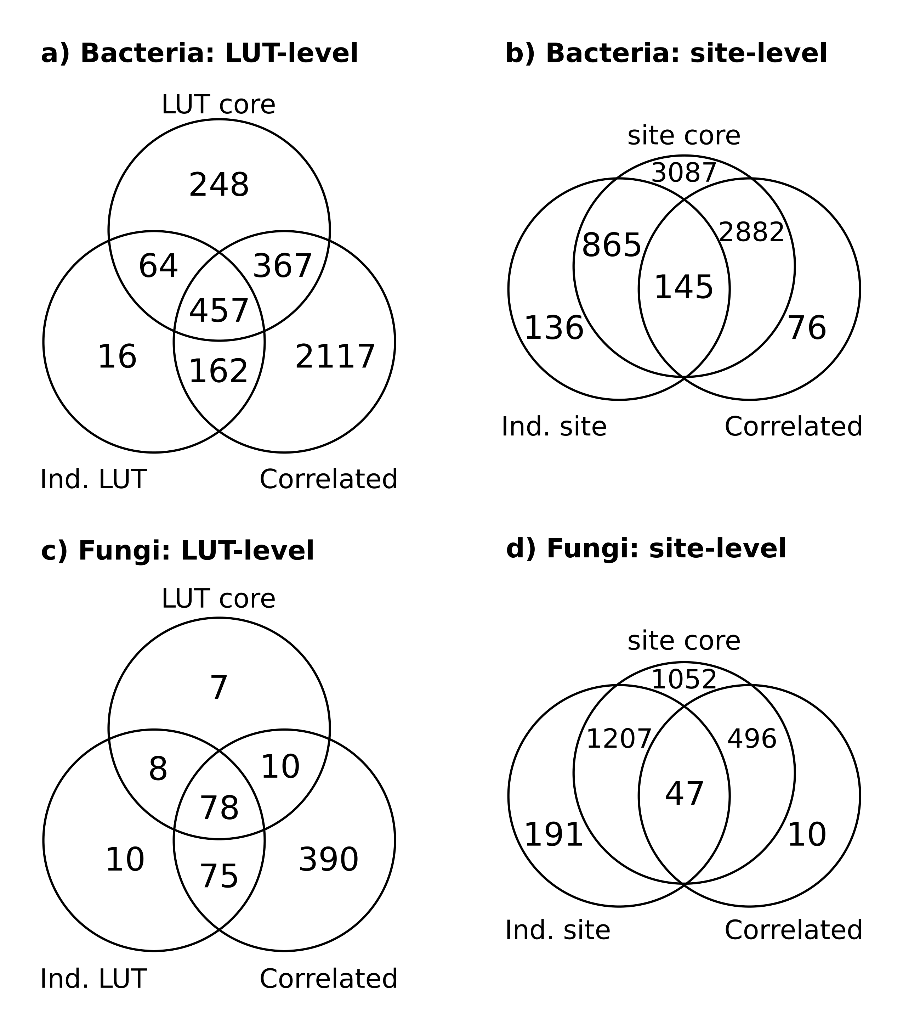


ind. site

ind. site

ind. env.

LUT core

ind. env.

ind. env.

ind. env.

ind. LUT

ind. LUT

site core

site core

LUT core

Figure S2: Venn diagramm depicting the OTUs that are shared between different OTU partitions. Bacterial (a, b) and fungal (c, d) OTU partitions are shown. Indicative and core OTUs were defined at two levels, i.e., land-use type (a, c) and site (b, d). Site core (sc) OTUs were defined as OTUs occurring in at least 80% of samples form a site, land-use type (LUT) cores (lc) as OTUs that were site cores of at least 80% of the sites from a land-use type. Indicative (ind.) OTUs were based on indicator species analysis (IndVal > 0.8), and environmental-factor-indicative-OTUs represent OTUs that revealed correlations to an environmental factor (|rho| > 0.4).


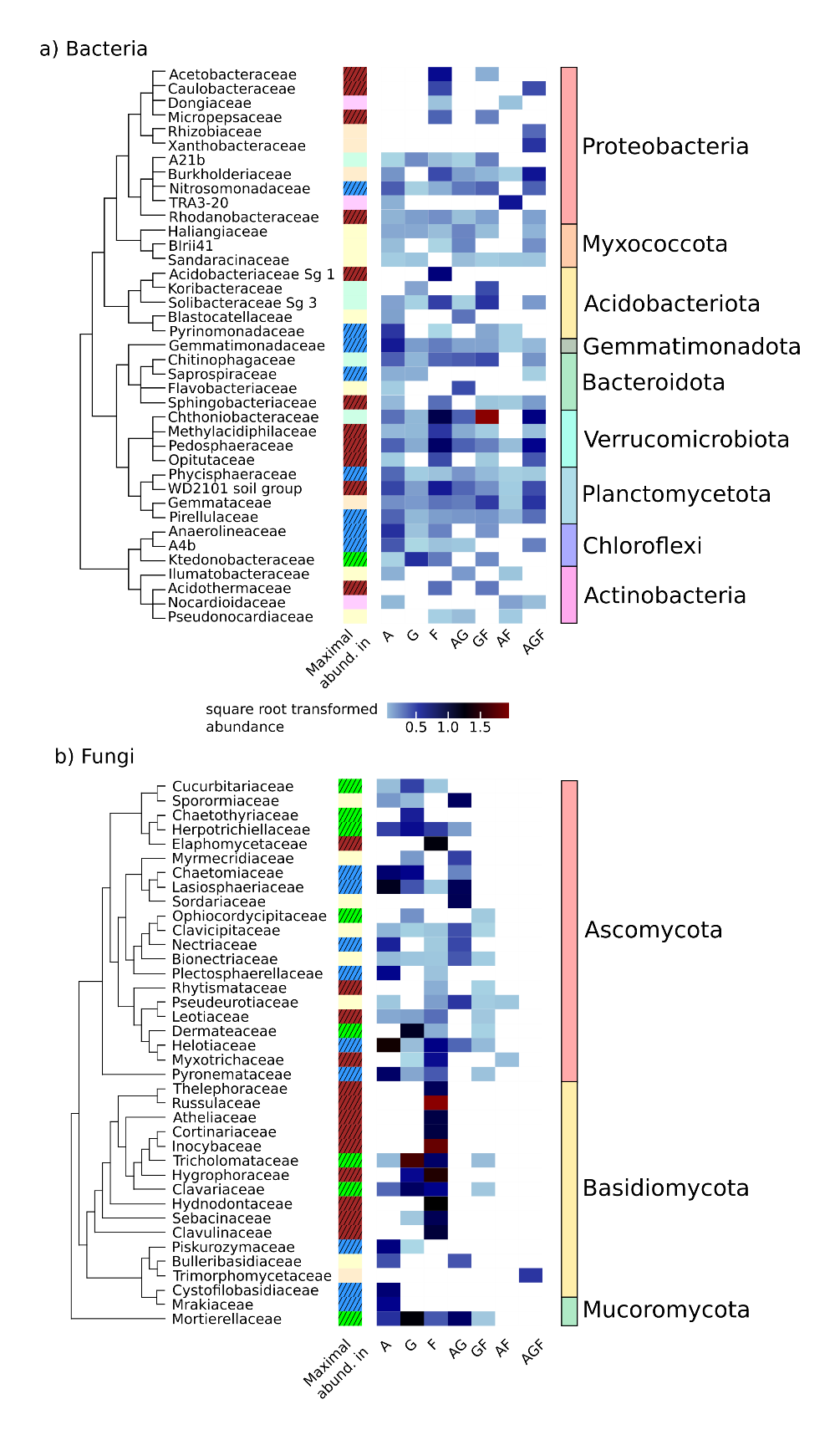


Figure S3: Distribution of most abundant bacterial (a) and fungal (b) families among land-use types. The same families as in Figure 4 are shown (see there for the selection criteria). Here, families were grouped according to their taxonomy. Only few closely related families shared similar distributions among land-use types. For bacteria, these include for instance the three families of the phylum Myxococcota, Acetobacteraceae, Caulobacteraceae, Dongiaceae, and Micropepsaceae of the Proteobacteria, as well as Anaerolineaceae and A4b of Chloroflexi. For fungi, these include for instance Thelephoraceae and Russulaceae, Cortinariaceae and Inocybaceae, and Cystofilobasidiaceae and Mrakiaceae.

Relationships of bacterial phyla are based on Coleman et al. 2021, fungal clustering is based on phylogenetic analyses by Li et al. (2021), as well as Hongsanan et al. (2017) for relationships of families belonging to Sordariomycetes, Johnston et al. (2019) for families belonging to Leotiomycetes, and Zhao et al. 2017 for families belonging to Agaricales.

Light blue indicates low, dark blue middle, and brown high relative abundances. White areas represent absences of families in an area of the ternary plot. The area in which a family has its highest abundance is indicated by the following color code (Maximal abund.): blue (A), green (G), brown (F), yellow (AG), light green (GF), pink (AF), and orange (AGF). Highest abundances in a single land-use type are indicated by black hatching.

**References:**

Coleman, G. A., Davín, A. A., Mahendrarajah, T. A., Szánthó, L. L., Spang, A., Hugenholtz, P., ... & Williams, T. A. (2021). A rooted phylogeny resolves early bacterial evolution. *Science*, *372*(6542).

Hongsanan, S., Maharachchikumbura, S. S., Hyde, K. D., Samarakoon, M. C., Jeewon, R., Zhao, Q., ... & Bahkali, A. H. (2017). An updated phylogeny of Sordariomycetes based on phylogenetic and molecular clock evidence. *Fungal diversity*, *84*(1), 25-41.

Johnston, P. R., Quijada, L., Smith, C. A., Baral, H. O., Hosoya, T., Baschien, C., ... & Townsend, J. P. (2019). A multigene phylogeny toward a new phylogenetic classification of Leotiomycetes. *IMA fungus*, *10*(1), 1-22.

Li, Y., Steenwyk, J. L., Chang, Y., Wang, Y., James, T. Y., Stajich, J. E., ... & Rokas, A. (2021). A genome-scale phylogeny of the kingdom Fungi. *Current Biology*, *31*(8), 1653-1665.

Zhao, R. L., Li, G. J., Sanchez-Ramirez, S., Stata, M., Yang, Z. L., Wu, G., ... & Hyde, K. D. (2017). A six-gene phylogenetic overview of Basidiomycota and allied phyla with estimated divergence times of higher taxa and a phyloproteomics perspective. *Fungal Diversity*, *84*(1), 43-74.

Table S1: Summary of environmental factors and alpha diversity at the ten sites of each land-use type.

|  |  | Arable land | | |  | Grassland | | |  | Forest | | |  | ANOVA^1)^ | |  |
| --- | --- | --- | --- | --- | --- | --- | --- | --- | --- | --- | --- | --- | --- | --- | --- | --- |
|  |  | mean | min | max |  | mean | min | max |  | mean | min | max |  | F | p-value | Pattern^2)^ |
| Site characteristics | |  |  |  |  |  |  |  |  |  |  |  |  |  |  |  |
|  | Elevation [masl] | 499.5 | 336.0 | 830.0 |  | 883.3 | 431.0 | 1915.0 |  | 967.6 | 505.0 | 1655.0 |  | 4.8 | 0.0165 | A<G=F |
|  | Clay [%] | 25.0 | 5.8 | 59.0 |  | 25.5 | 12.5 | 35.0 |  | 23.1 | 7.0 | 42.0 |  | 0.1 | 0.8943 | A=G=F |
|  | Silt [%] | 42.7 | 30.0 | 59.8 |  | 38.9 | 27.0 | 55.0 |  | 36.1 | 18.6 | 52.0 |  | 1.0 | 0.3993 | A=G=F |
|  | Sand [%] | 32.3 | 11.0 | 54.0 |  | 35.5 | 12.0 | 50.8 |  | 40.8 | 17.5 | 71.0 |  | 0.7 | 0.4927 | A=G=F |
|  | Soil skeleton [%] | 2.4 | 0.0 | 4.9 |  | 2.8 | 0.0 | 11.3 |  | 3.9 | 0.0 | 11.2 |  | 0.6 | 0.5640 | A=G=F |
|  | MAT^3)^ [°C] | 8.9 | 3.9 | 11.0 |  | 5.4 | -2.3 | 11.0 |  | 7.7 | 0.2 | 12.3 |  | 2.5 | 0.1023 | A=G=F |
|  | MAP^4)^ [mm] | 1154.2 | 905.0 | 1838.0 |  | 1510.4 | 1090.0 | 1979.0 |  | 1191.3 | 528.0 | 2140.0 |  | 2.9 | 0.0711 | A=G=F |
| Yearly measurements | |  |  |  |  |  |  |  |  |  |  |  |  |  |  |  |
|  | pH | 6.6 | 5.6 | 7.5 |  | 5.2 | 3.8 | 6.3 |  | 4.8 | 3.3 | 6.9 |  | 10.6 | 0.0004 | A>G=F |
|  | C_tot_ [%] | 2.4 | 1.1 | 4.5 |  | 4.1 | 2.6 | 7.0 |  | 7.3 | 2.4 | 18.3 |  | 7.7 | 0.0023 | A=G<F |
|  | C_org_ [%] | 2.2 | 1.1 | 3.4 |  | 4.1 | 2.6 | 7.0 |  | 7.3 | 2.4 | 18.3 |  | 8.3 | 0.0015 | A=G<F |
|  | N_tot_ [%] | 0.2 | 0.1 | 0.5 |  | 0.4 | 0.3 | 0.7 |  | 0.4 | 0.2 | 1.1 |  | 3.2 | 0.0549 | A=G=F |
|  | C/N | 9.0 | 6.7 | 11.2 |  | 9.6 | 7.4 | 12.5 |  | 17.8 | 11.3 | 27.5 |  | 40.0 | < 0.0001 | A=G<F |
|  | Bulk density [kg dm^-3^] | 1.2 | 0.6 | 1.5 |  | 1.0 | 0.7 | 1.2 |  | 0.7 | 0.2 | 1.2 |  | 14.9 | < 0.0001 | A>G>F |
|  | C_mic_ [mg C kg(dry soil)^-1^]^5)^ | 647.5 | 229.9 | 1247.0 |  | 1574.1 | 986.4 | 2521.5 |  | 2266.4 | 563.6 | 7727.3 |  | 9.6 | 0.0007 | A<G=F |
|  | DNA [mg kg^-1^] | 21.2 | 12.3 | 41.7 |  | 41.4 | 18.0 | 70.0 |  | 50.2 | 15.0 | 127.0 |  | 10.8 | 0.0004 | A<G=F |
| Bacteria | |  |  |  |  |  |  |  |  |  |  |  |  |  |  |  |
|  | OTU richness | 2405.3 | 1653.9 | 3036.3 |  | 2295.4 | 1347.3 | 2768.2 |  | 1833.1 | 1112.8 | 2735.2 |  | 5.5 | 0.0096 | A=G>F |
|  | Simpson evenness | 0.156 | 0.062 | 0.203 |  | 0.108 | 0.029 | 0.224 |  | 0.082 | 0.012 | 0.165 |  | 11.8 | 0.0002 | A>G=F |
|  | Inverse Simpson | 377.0 | 116.5 | 561.7 |  | 253.4 | 69.0 | 568.3 |  | 151.8 | 21.6 | 428.7 |  | 13.2 | 0.0001 | A>G=F |
| Fungi | |  |  |  |  |  |  |  |  |  |  |  |  |  |  |  |
|  | OTU richness | 400.7 | 277.8 | 553.9 |  | 426.0 | 219.8 | 730.9 |  | 406.6 | 282.2 | 625.9 |  | 0.4 | 0.6853 | A=G=F |
|  | Simpson evenness | 0.096 | 0.023 | 0.185 |  | 0.083 | 0.012 | 0.180 |  | 0.050 | 0.008 | 0.105 |  | 7.5 | 0.0026 | A=G>F |
|  | Inverse Simpson | 39.3 | 8.3 | 96.3 |  | 37.4 | 4.2 | 90.5 |  | 20.3 | 2.7 | 47.6 |  | 5.5 | 0.0100 | A=G>F |

^1)^ One-way ANOVA for site characteristics, ANOVA with repeated measurement design including site as a random factor for yearly measured properties;

^2)^ Significant differences of pairwise tests between land-use types (p < 0.05): A - arable land, G - grassland, F - forest;

^3)^ MAT: mean annual temperature;

^4)^ MAP: mean annual precipitation;

^5)^ C_mic_: microbial carbon (carbon content based on chloroform fumigation extraction).

Table S2: Spearman correlations of entire communities with those composed of site-core OTUs (sc-OTUs). OTUs being detected in at least 12 of the 15 samples from a site were classified as sc-OTUs.

|  |  | Bacteria | |  | Fungi | |
| --- | --- | --- | --- | --- | --- | --- |
|  |  | rho | p-value |  | rho | p-value |
| Alpha-diversity | |  |  |  |  |  |
|  | OTU richness | 0.99 | < 2.2e-16 |  | 0.97 | < 2.2e-16 |
|  | Simpson evenness | 1.00 | < 2.2e-16 |  | 0.98 | < 2.2e-16 |
|  | Inverse Simpson | 1.00 | < 2.2e-16 |  | 0.98 | < 2.2e-16 |
| Beta diversity | |  |  |  |  |  |
|  | Jaccard | 1.00 | 0.0001 |  | 1.00 | 0.0001 |
|  | Bray-Curtis | 1.00 | 0.0001 |  | 1.00 | 0.0001 |

Table S3: Environmental influences on bacterial communities in three land-use types as assessed by nested PERMANOVA. Model selection used AICc as selection criteria.

| Arable land | | | |  | Grassland | | | |  |  | Forest | | | |
| --- | --- | --- | --- | --- | --- | --- | --- | --- | --- | --- | --- | --- | --- | --- |
| Factor | PF^1)^ | √CV^2)^ | p-value |  | Factor | PF^1)^ | √CV^2)^ | p-value |  |  | Factor | PF^1)^ | √CV^2)^ | p-value |
| pH | 7.3 | 0.18 | 0.0001 |  | pH | 8.3 | 0.21 | 0.0001 |  |  | pH | 7.2 | 0.27 | 0.0001 |
| MAT^3)^ | 3.0 | 0.12 | 0.0001 |  | C_org_ | 3.0 | 0.11 | 0.0001 |  |  | Clay | 1.8 | 0.11 | 0.0401 |
| Soil skeleton | 1.9 | 0.07 | 0.0001 |  | MAT^3)^ | 1.6 | 0.11 | 0.0077 |  |  | MAP^4)^ | 1.5 | 0.12 | 0.1326 |
| C_org_ | 1.6 | 0.07 | 0.0003 |  | Clay | 1.4 | 0.07 | 0.0302 |  |  | Year | 1.9 | 0.05 | 0.0001 |
| Silt | 1.2 | 0.05 | 0.0760 |  | C/N-ratio | 1.6 | 0.06 | 0.0123 |  |  | Site | 24.7 | 0.34 | 0.0001 |
| Year | 1.9 | 0.06 | 0.0001 |  | Year | 3.0 | 0.07 | 0.0001 |  |  | Residuals |  | 0.15 |  |
| Crop | 1.6 | 0.08 | 0.0002 |  | Site | 10.3 | 0.23 | 0.0001 |  |  |  |  |  |  |
| Site | 5.3 | 0.21 | 0.0001 |  | Residuals |  | 0.14 |  |  |  |  |  |  |  |
| Residuals |  | 0.16 |  |  |  |  |  |  |  |  |  |  |  |  |
|  |  |  |  |  |  |  |  |  |  |  |  |  |  |  |

^1)^ PF: Pseudo-F;
^2)^√CV: square root of component of variation;
^3)^ MAT: mean annual temperature;
^4)^ MAP: Mean annual precipitation.

Table S4: Environmental influences on fungal communities in three land-use types as assessed by nested PERMANOVA. Model selection used AICc as selection criteria.

| Arable land | | | |  | | Grassland | | | | | | | | |  |  | | Forest | | | | | | | |
| --- | --- | --- | --- | --- | --- | --- | --- | --- | --- | --- | --- | --- | --- | --- | --- | --- | --- | --- | --- | --- | --- | --- | --- | --- | --- |
| Factor | PF^1)^ | √CV^2)^ | p-value |  | | Factor | | PF^1)^ | | √CV | | p-value | | |  |  | | Factor | | PF^1)^ | | √CV^2)^ | | p-value | |
| pH | 1.7 | 0.11 | 0.0050 |  | | pH | | 4.5 | | 0.23 | | 0.0001 | | |  |  | | pH | | 1.4 | | 0.13 | | 0.2000 | |
| Year | 1.4 | 0.08 | 0.0025 |  | | Altitude | | 2.3 | | 0.20 | | 0.0009 | | |  |  | | Site | | 13.6 | | 0.59 | | 0.0001 | |
| Crop | 1.5 | 0.15 | 0.0007 |  | | Clay | | 1.5 | | 0.09 | | 0.0553 | | |  |  | | Residuals | |  | | 0.36 | |  | |
| Site | 6.6 | 0.38 | 0.0001 |  | | Soil skeleton | | 1.3 | | 0.09 | | 0.1192 | | |  |  | |  | |  | |  | |  | |
| Residuals |  | 0.31 |  |  | | C_org_ | | 1.6 | | 0.18 | | 0.0413 | | |  |  | |  | |  | |  | |  | |
|  |  |  |  |  | | Year | | 2.3 | | 0.09 | | 0.0001 | | |  |  | |  | |  | |  | |  | |
|  |  |  |  |  | | Site | | 10.2 | | 0.38 | | 0.0001 | | |  |  | |  | |  | |  | |  | |
|  |  |  |  |  | Residuals | |  | | 0.23 | |  | |  |  | | |  | |  | |  | |  | |  |

^1)^ PF: Pseudo-F;
^2)^ √CV: square root of component of variation.

Table S5: Bacterial OTUs with the strongest negative and positive correlations to an environmental factor along with their taxonomic assignment. The 20 strongest correlations are shown for negative and positive correlations.

|  | OTUID | Factor^1)^ | Rho | Phylum | Class | Order | Family | Genus |
| --- | --- | --- | --- | --- | --- | --- | --- | --- |
| positively correlated | bOTU 57 | pH | 0.91 | Acidobacteria | Sg 6 | unclassified | unclassified | unclassified |
|  | bOTU 16256 | pH | 0.90 | Acidobacteria | Sg 6 | unclassified | unclassified | unclassified |
|  | bOTU 547 | pH | 0.90 | Acidobacteria | Sg 6 | unclassified | unclassified | unclassified |
|  | bOTU 214 | pH | 0.89 | Proteobacteria | Gammaproteobacteria | Steroidobacterales | Steroidobacteraceae | unclassified |
|  | bOTU 4698 | pH | 0.89 | Acidobacteria | Sg 6 | unclassified | unclassified | unclassified |
|  | bOTU 144 | pH | 0.88 | Bacteroidetes | Bacteroidia | Cytophagales | Microscillaceae | *Chryseolinea* |
|  | bOTU 65 | pH | 0.88 | Acidobacteria | Sg 6 | unclassified | unclassified | unclassified |
|  | bOTU 487 | pH | 0.88 | Acidobacteria | Sg 6 | unclassified | unclassified | unclassified |
|  | bOTU 240 | pH | 0.87 | Proteobacteria | Gammaproteobacteria | Betaproteobacteriales | Nitrosomonadaceae | Ellin6067 |
|  | bOTU 223 | pH | 0.87 | Bacteroidetes | Bacteroidia | Cytophagales | Microscillaceae | unclassified |
|  | bOTU 2023 | pH | 0.86 | Acidobacteria | Blastocatellia (Sg 4) | Blastocatellales | Blastocatellaceae | *Stenotrophobacter* |
|  | bOTU 647 | pH | 0.86 | Chloroflexi | Anaerolineae | SBR1031 | A4b | unclassified |
|  | bOTU 682 | pH | 0.86 | Acidobacteria | Sg 6 | unclassified | unclassified | unclassified |
|  | bOTU 114 | pH | 0.86 | Acidobacteria | Sg 6 | unclassified | unclassified | unclassified |
|  | bOTU 4 | pH | 0.86 | Acidobacteria | Blastocatellia (Sg 4) | Blastocatellales | Blastocatellaceae | unclassified |
|  | bOTU 120 | pH | 0.86 | Verrucomicrobia | Verrucomicrobiae | Verrucomicrobiales | Verrucomicrobiaceae | unclassified |
|  | bOTU 7542 | pH | 0.86 | Bacteroidetes | Bacteroidia | Chitinophagales | Chitinophagaceae | *Terrimonas* |
|  | bOTU 299 | pH | 0.85 | Acidobacteria | Sg 6 | unclassified | unclassified | unclassified |
|  | bOTU 391 | pH | 0.85 | Actinobacteria | Thermoleophilia | Gaiellales | Gaiellaceae | *Gaiella* |
|  | bOTU 96 | pH | 0.85 | Planctomycetes | Planctomycetacia | Pirellulales | Pirellulaceae | *Pirellula* |
| negatively correlated | bOTU 596 | pH | -0.82 | Verrucomicrobia | Verrucomicrobiae | S-BQ2-57 soil group | unclassified | unclassified |
|  | bOTU 5377 | pH | -0.82 | Acidobacteria | Acidobacteriia | Solibacterales | Solibacteraceae (Sg 3) | *Bryobacter* |
|  | bOTU 9645 | pH | -0.82 | Acidobacteria | Acidobacteriia | Acidobacteriales | unclassified | unclassified |
|  | bOTU 215 | pH | -0.82 | Proteobacteria | Deltaproteobacteria | RCP2-54 | unclassified | unclassified |
|  | bOTU 961 | pH | -0.83 | Acidobacteria | Acidobacteriia | Solibacterales | Solibacteraceae (Sg 3) | *Bryobacter* |
|  | bOTU 60 | pH | -0.83 | Acidobacteria | Acidobacteriia | Sg 2 | unclassified | unclassified |
|  | bOTU 712 | pH | -0.83 | Proteobacteria | Alphaproteobacteria | Micropepsales | Micropepsaceae | unclassified |
|  | bOTU 71 | pH | -0.83 | Verrucomicrobia | Verrucomicrobiae | Pedosphaerales | Pedosphaeraceae | unclassified |
|  | bOTU 50 | pH | -0.84 | Acidobacteria | Acidobacteriia | Sg 2 | unclassified | unclassified |
|  | bOTU 514 | pH | -0.84 | Proteobacteria | Gammaproteobacteria | Incertae sedis | unknown family | *Acidibacter* |
|  | bOTU 163 | pH | -0.84 | Acidobacteria | Acidobacteriia | Acidobacteriales | Koribacteraceae | Cand.^3)^ Koribacter |
|  | bOTU 1665 | pH | -0.84 | Verrucomicrobia | Verrucomicrobiae | Pedosphaerales | Pedosphaeraceae | unclassified |
|  | bOTU 70 | pH | -0.84 | Proteobacteria | Alphaproteobacteria | Rhizobiales | Beijerinckiaceae | *Roseiarcus* |
|  | bOTU 44 | pH | -0.84 | Verrucomicrobia | Verrucomicrobiae | Chthoniobacterales | Xiphinematobacteraceae | Cand. Xiphinematobacter |
|  | bOTU 452 | pH | -0.85 | Planctomycetes | Phycisphaerae | Tepidisphaerales | WD2101 soil group | unclassified |
|  | bOTU 15161 | pH | -0.86 | Acidobacteria | Acidobacteriia | Acidobacteriales | Acidobacteriaceae (Sg 1) | *Occallatibacter* |
|  | bOTU 1433 | pH | -0.87 | Acidobacteria | Acidobacteriia | Acidobacteriales | Koribacteraceae | Cand.^3)^ Koribacter |
|  | bOTU 283 | pH | -0.87 | Acidobacteria | Acidobacteriia | Acidobacteriales | unclassified | unclassified |
|  | bOTU 396 | pH | -0.88 | Acidobacteria | Acidobacteriia | Acidobacteriales | unclassified | unclassified |
|  | bOTU 9919 | pH | -0.88 | Acidobacteria | Acidobacteriia | Sg^2)^ 2 | unclassified | unclassified |
|  | bOTU 151 | pH | -0.88 | Actinobacteria | Actinobacteria | Frankiales | Acidothermaceae | *Acidothermus* |
|  | bOTU 35 | pH | -0.92 | Acidobacteria | Acidobacteriia | Acidobacteriales | unclassified | unclassified |

^1)^ Environmental factors include: altitude, clay, silt, sand, soil skeleton, soil pH, total and organic carbon, total nitrogen, C/N-ratio, bulk density, mean annual temperature and mean annual precipitation;
^2)^ Sg: subgroup;
^3)^ Cand. Candidatus.

Table S6: Fungal OTUs with the strongest negative and positive correlations to an environmental factor along with their taxonomic assignment. The 20 strongest correlations are shown for negative and positive correlations.

|  | OTUID | Factor^1)^ | Rho | Phylum | Class | Order | Family | Genus |
| --- | --- | --- | --- | --- | --- | --- | --- | --- |
| positively correlated | fOTU 228 | C/N | 0.75 | Ascomycota | Leotiomycetes | Helotiales | Myxotrichaceae | *Oidiodendron* |
|  | fOTU 647 | pH | 0.74 | Ascomycota | unclassified | unclassified | unclassified | unclassified |
|  | fOTU 1017 | C/N | 0.73 | Basidiomycota | Microbotryomycetes | Leucosporidiales | unclassified | unclassified |
|  | fOTU 1091 | C/N | 0.72 | Ascomycota | Leotiomycetes | Helotiales | Myxotrichaceae | *Oidiodendron* |
|  | fOTU 361 | C/N | 0.71 | Ascomycota | Saccharomycetes | Saccharomycetales | unclassified | unclassified |
|  | fOTU 365 | pH | 0.71 | Ascomycota | Dothideomycetes | Pleosporales | Phaeosphaeriaceae | unclassified |
|  | fOTU 7764 | C/N | 0.69 | Ascomycota | Leotiomycetes | Thelebolales | Pseudeurotiaceae | *Geomyces* |
|  | fOTU 12 | pH | 0.69 | Ascomycota | Leotiomycetes | Helotiales | Helotiaceae | *Tetracladium* |
|  | fOTU 2496 | C/N | 0.69 | Ascomycota | Eurotiomycetes | Chaetothyriales | Herpotrichiellaceae | *Cladophialophora* |
|  | fOTU 10774 | C/N | 0.69 | Mortierellomycota | Mortierellomycetes | Mortierellales | Mortierellaceae | *Mortierella* |
|  | fOTU 12 | Bulk density | 0.68 | Ascomycota | Leotiomycetes | Helotiales | Helotiaceae | *Tetracladium* |
|  | fOTU 354 | C/N | 0.67 | Ascomycota | Eurotiomycetes | Chaetothyriales | Herpotrichiellaceae | *Cladophialophora* |
|  | fOTU 718 | C/N | 0.65 | Ascomycota | unclassified | unclassified | unclassified | unclassified |
|  | fOTU 1804 | C/N | 0.65 | Ascomycota | Leotiomycetes | Helotiales | Vibrisseaceae | *Phialocephala* |
|  | fOTU 183 | pH | 0.65 | Ascomycota | Sordariomycetes | Sordariales | Lasiosphaeriaceae | *Podospora* |
|  | fOTU 236 | C/N | 0.65 | Ascomycota | Leotiomycetes | Helotiales | Helotiaceae | *Meliniomyces* |
|  | fOTU 39 | Bulk density | 0.65 | Ascomycota | Sordariomycetes | Sordariales | Chaetomiaceae | unclassified |
|  | fOTU 70 | Bulk density | 0.64 | Ascomycota | Sordariomycetes | Glomerellales | Plectosphaerellaceae | *Gibellulopsis* |
|  | fOTU 764 | C/N | 0.64 | Ascomycota | Leotiomycetes | Helotiales | Myxotrichaceae | *Oidiodendron* |
|  | fOTU 441 | C/N | 0.64 | Ascomycota | Leotiomycetes | Helotiales | Helotiales i.s.^2)^ | *Cadophora* |
| negatively correlated | fOTU 79 | C/N | -0.65 | Ascomycota | Sordariomycetes | Myrmecridiales | Myrmecridiaceae | *Myrmecridium* |
|  | fOTU 36 | Corg | -0.65 | Ascomycota | Sordariomycetes | Hypocreales | Nectriaceae | unclassified |
|  | fOTU 803 | Corg | -0.65 | Ascomycota | Sordariomycetes | Glomerellales | Plectosphaerellaceae | *Plectosphaerella* |
|  | fOTU 20 | Corg | -0.65 | Ascomycota | Leotiomycetes | Helotiales | Helotiaceae | *Tetracladium* |
|  | fOTU 1091 | Bulk density | -0.65 | Ascomycota | Leotiomycetes | Helotiales | Myxotrichaceae | *Oidiodendron* |
|  | fOTU 147 | C/N | -0.66 | Ascomycota | unclassified | unclassified | unclassified | unclassified |
|  | fOTU 56 | C/N | -0.66 | Ascomycota | Sordariomycetes | Glomerellales | Plectosphaerellaceae | *Plectosphaerella* |
|  | fOTU 1898 | Corg | -0.66 | Ascomycota | Sordariomycetes | Sordariales | Lasiosphaeriaceae | unclassified |
|  | fOTU 122 | C/N | -0.67 | Ascomycota | Leotiomycetes | Thelebolales | Pseudeurotiaceae | *Pseudeurotium* |
|  | fOTU 1898 | Ctot | -0.67 | Ascomycota | Sordariomycetes | Sordariales | Lasiosphaeriaceae | unclassified |
|  | fOTU 12 | Corg | -0.67 | Ascomycota | Leotiomycetes | Helotiales | Helotiaceae | *Tetracladium* |
|  | fOTU 12 | C/N | -0.68 | Ascomycota | Leotiomycetes | Helotiales | Helotiaceae | *Tetracladium* |
|  | fOTU 26 | C/N | -0.68 | Ascomycota | Sordariomycetes | Hypocreales | Nectriaceae | *Fusarium* |
|  | fOTU 4641 | C/N | -0.68 | Mortierellomycota | Mortierellomycetes | Mortierellales | Mortierellaceae | *Mortierella* |
|  | fOTU 39 | Corg | -0.68 | Ascomycota | Sordariomycetes | Sordariales | Chaetomiaceae | unclassified |
|  | fOTU 164 | C/N | -0.69 | Ascomycota | Sordariomycetes | Sordariales | Lasiosphaeriaceae | unclassified |
|  | fOTU 70 | Corg | -0.69 | Ascomycota | Sordariomycetes | Glomerellales | Plectosphaerellaceae | *Gibellulopsis* |
|  | fOTU 62 | C/N | -0.69 | Ascomycota | Sordariomycetes | Hypocreales | Bionectriaceae | *Clonostachys* |
|  | fOTU 20 | C/N | -0.69 | Ascomycota | Leotiomycetes | Helotiales | Helotiaceae | *Tetracladium* |
|  | fOTU 14 | C/N | -0.71 | Ascomycota | Sordariomycetes | Sordariales | Sordariaceae | unclassified |

^1)^ environmental factors include: altitude, clay, silt, sand, soil skeleton, soil pH, total and organic carbon, total nitrogen, C/N-ratio, bulk density, mean annual temperature, and mean annual precipitation;
^2)^ i.s.: incertae sedis

Table S7: Number of land-use-indicative bacterial OTUs grouped by family. All families containing at least one indicative OTU, as well as families represented in Figure 4c are shown.

|  | Number of OTUs | | | indicative and core OTUs (all indicative OTUs)^1)^ | | | | | | Figure 4d | |
| --- | --- | --- | --- | --- | --- | --- | --- | --- | --- | --- | --- |
| Family | Total | site-core | Ind. | A | AG | G | AF | FG | F | Cluster | Max. Abund |
| Pedosphaeraceae | 403 | 255 | 36 | 4(5) | 7(11) | 3(3) | 0(0) | 1(12) | 3(5) | VII | F |
| WD2101 soil group | 223 | 151 | 34 | 2(5) | 9(18) | 1(2) | 0(0) | 1(4) | 1(5) | VII | F |
| Chitinophagaceae | 236 | 130 | 31 | 4(10) | 3(12) | 2(2) | 0(0) | 1(4) | 1(3) | V | FG |
| Chthoniobacteraceae | 223 | 155 | 23 | 1(1) | 4(12) | 0(1) | 0(0) | 2(5) | 3(4) | III | FG |
| Gemmataceae | 696 | 318 | 20 | 0(0) | 2(5) | 1(1) | 0(0) | 0(14) | 0(0) | V | AGF |
| Solibacteraceae Sg 3 | 111 | 81 | 17 | 0(0) | (0)2 | 0(0) | 0(0) | (3)12 | (1)3 | VII | FG |
| Gemmatimonadaceae | 271 | 180 | 17 | 5(9) | 3(5) | 0(0) | 0(0) | 0(2) | 1(1) | VI | A |
| Pirellulaceae | 302 | 152 | 15 | 4(5) | 1(8) | 0(0) | 0(0) | 0(2) | 0(0) | V | A |
| Burkholderiaceae | 129 | 80 | 14 | 1(2) | 4(7) | 0(0) | 0(0) | 0(1) | 3(4) | VII | AGF |
| Xanthomonadaceae | 36 | 22 | 12 | 1(2) | 2(8) | 0(0) | 0(1) | 0(1) | 0(0) |  |  |
| SC-I-84 | 51 | 44 | 10 | 1(1) | 2(4) | 2(2) | 0(0) | 0(3) | 0(0) |  |  |
| Acidobacteriaceae Sg1 | 26 | 23 | 9 | 0(0) | 0(0) | 0(0) | 0(0) | 0(1) | 6(8) | VII | F |
| Haliangiaceae | 201 | 100 | 9 | 2(2) | 0(5) | 0(0) | 0(0) | 0(2) | 0(0) | V | AG |
| Acetobacteraceae | 42 | 25 | 8 | 0(0) | 0(0) | 0(0) | 0(0) | 2(2) | 4(6) | VII | F |
| Sphingobacteriaceae | 65 | 32 | 8 | 1(1) | 0(2) | 0(0) | 0(0) | 0(1) | 2(4) | VII | F |
| Anaerolineaceae | 134 | 87 | 8 | 4(4) | 0(3) | 0(0) | 0(0) | 0(1) | 0(0) | VI | A |
| Acidothermaceae | 18 | 15 | 7 | 0(0) | 0(0) | 0(0) | 0(0) | 2(3) | 2(4) | VII | F |
| Methylacidiphilaceae | 81 | 31 | 7 | 0(0) | 0(0) | 0(0) | 0(0) | 0(2) | 2(5) | VII | F |
| Phycisphaeraceae | 254 | 98 | 7 | 0(0) | 2(7) | 0(0) | 0(0) | 0(0) | 0(0) | VI | A |
| Nitrosomonadaceae | 79 | 67 | 7 | 0(0) | 1(4) | 0(0) | 0(1) | 0(2) | 0(0) | V | A |
| Caulobacteraceae | 26 | 21 | 6 | 0(0) | 1(1) | 0(0) | 0(0) | 0(0) | 3(5) | VII | F |
| A21b | 23 | 18 | 6 | 0(0) | 0(1) | 1(1) | 0(0) | 1(4) | 0(0) | III | FG |
| Beijerinckiaceae | 21 | 10 | 6 | 0(0) | 0(4) | 0(0) | 0(0) | 0(1) | 1(1) |  |  |
| Polyangiaceae | 79 | 44 | 6 | 0(0) | 1(4) | 0(0) | 0(0) | 0(2) | 0(0) |  |  |
| A4b | 138 | 88 | 5 | 3(5) | 0(0) | 0(0) | 0(0) | 0(0) | 0(0) | VI | A |
| Pyrinomonadaceae | 28 | 22 | 5 | 4(4) | 0(0) | 0(0) | 0(0) | 0(1) | 0(0) | VI | A |
| Flavobacteriaceae | 52 | 27 | 5 | 0(0) | 3(5) | 0(0) | 0(0) | 0(0) | 0(0) | V | AG |
| Koribacteraceae | 11 | 11 | 5 | 0(0) | 0(0) | 2(2) | 0(0) | 2(3) | 0(0) | III | FG |
| Nocardioidaceae | 28 | 16 | 5 | 1(1) | 0(4) | 0(0) | 0(0) | 0(0) | 0(0) | II | AF |
| 67-14 | 56 | 32 | 5 | 2(2) | 1(3) | 0(0) | 0(0) | 0(0) | 0(0) |  |  |
| CPla-3_termite_group | 39 | 29 | 5 | 0(0) | 2(2) | 0(0) | 0(0) | 0(2) | 1(1) |  |  |
| JG30-KF-CM45 | 86 | 45 | 5 | 2(4) | 1(1) | 0(0) | 0(0) | 0(0) | 0(0) |  |  |
| Verrucomicrobiaceae | 83 | 38 | 5 | 1(2) | 1(3) | 0(0) | 0(0) | 0(0) | 0(0) |  |  |
| Micropepsaceae | 16 | 15 | 4 | 0(0) | 0(0) | 0(0) | 0(0) | 2(4) | 0(0) | VII | F |
| Opitutaceae | 48 | 31 | 4 | 0(0) | 1(2) | 0(0) | 0(0) | 0(1) | 0(1) | VII | F |
| Ilumatobacteraceae | 13 | 11 | 4 | 0(1) | 3(3) | 0(0) | 0(0) | 0(0) | 0(0) | V | AG |
| AKYH767 | 66 | 35 | 4 | 0(0) | 4(4) | 0(0) | 0(0) | 0(0) | 0(0) |  |  |
| Fimbriimonadaceae | 62 | 34 | 4 | 2(2) | 0(1) | 0(0) | 0(0) | 0(0) | 0(1) |  |  |
| Gaiellaceae | 18 | 15 | 4 | 0(0) | 2(4) | 0(0) | 0(0) | 0(0) | 0(0) |  |  |
| Geobacteraceae | 35 | 23 | 4 | 0(0) | 1(4) | 0(0) | 0(0) | 0(0) | 0(0) |  |  |
| Sphingomonadaceae | 29 | 25 | 4 | 0(2) | 1(1) | 0(0) | 0(0) | 0(0) | 0(1) |  |  |
| Blastocatellaceae | 18 | 17 | 3 | 0(1) | 0(1) | 0(0) | 0(0) | 0(1) | 0(0) | V | AG |
| TRA3-20 | 17 | 15 | 3 | 1(2) | 0(1) | 0(0) | 0(0) | 0(0) | 0(0) | II | AF |
| Bdellovibrionaceae | 265 | 50 | 3 | 0(0) | 0(2) | 0(0) | 0(0) | 0(0) | 0(1) |  |  |
| Fibrobacteraceae | 26 | 11 | 3 | 0(0) | 1(2) | 0(0) | 0(0) | 0(1) | 0(0) |  |  |
| Iamiaceae | 19 | 13 | 3 | 0(0) | 2(3) | 0(0) | 0(0) | 0(0) | 0(0) |  |  |
| Isosphaeraceae | 76 | 24 | 3 | 0(0) | 0(0) | 0(0) | 0(1) | 1(1) | 1(1) |  |  |
| Microscillaceae | 73 | 40 | 3 | 0(0) | 1(1) | 0(0) | 0(0) | 0(2) | 0(0) |  |  |
| Solirubrobacteraceae | 44 | 23 | 3 | 0(0) | 0(1) | 0(0) | 0(0) | 0(2) | 0(0) |  |  |
| Steroidobacteraceae | 21 | 15 | 3 | 1(1) | 0(2) | 0(0) | 0(0) | 0(0) | 0(0) |  |  |
| Rhodanobacteraceae | 45 | 31 | 2 | 0(0) | 0(0) | 0(0) | 0(0) | 1(1) | 0(1) | VII | F |
| BIrii41 | 63 | 34 | 2 | 0(0) | 0(1) | 1(1) | 0(0) | 0(0) | 0(0) | V | AG |
| Pseudonocardiaceae | 14 | 8 | 2 | 0(0) | 0(2) | 0(0) | 0(0) | 0(0) | 0(0) | V | AG |
| Ktedonobacteraceae | 210 | 112 | 2 | 0(0) | 0(0) | 0(0) | 0(0) | 0(2) | 0(0) | IV | G |
| Bacillaceae | 14 | 4 | 2 | 0(0) | 2(2) | 0(0) | 0(0) | 0(0) | 0(0) |  |  |
| Caldilineaceae | 51 | 22 | 2 | 1(1) | 0(1) | 0(0) | 0(0) | 0(0) | 0(0) |  |  |
| Elsteraceae | 18 | 8 | 2 | 0(0) | 0(0) | 0(0) | 0(0) | 0(0) | 0(2) |  |  |
| Herpetosiphonaceae | 15 | 7 | 2 | 2(2) | 0(0) | 0(0) | 0(0) | 0(0) | 0(0) |  |  |
| Hymenobacteraceae | 26 | 8 | 2 | 0(0) | 1(2) | 0(0) | 0(0) | 0(0) | 0(0) |  |  |
| Methylophilaceae | 12 | 8 | 2 | 1(1) | 0(1) | 0(0) | 0(0) | 0(0) | 0(0) |  |  |
| NS11-12 marine group | 40 | 9 | 2 | 0(0) | 1(2) | 0(0) | 0(0) | 0(0) | 0(0) |  |  |
| Solimonadaceae | 31 | 7 | 2 | 1(1) | 0(1) | 0(0) | 0(0) | 0(0) | 0(0) |  |  |
| Xiphinematobacteraceae | 14 | 10 | 2 | 0(0) | 0(0) | 0(0) | 0(0) | 1(1) | 1(1) |  |  |
| Dongiaceae | 8 | 6 | 1 | 0(0) | 0(0) | 0(0) | 0(1) | 0(0) | 0(0) | II | AF |
| Xanthobacteraceae | 16 | 16 | 1 | 0(0) | 0(0) | 0(0) | 0(0) | 1(1) | 0(0) | I | AGF |
| 37-13 | 22 | 14 | 1 | 0(0) | 1(1) | 0(0) | 0(0) | 0(0) | 0(0) |  |  |
| Aeromonadaceae | 1 | 1 | 1 | 0(0) | 0(1) | 0(0) | 0(0) | 0(0) | 0(0) |  |  |
| Amb-16S-1034 | 1 | 1 | 1 | 0(0) | 0(0) | 0(0) | 0(0) | 0(1) | 0(0) |  |  |
| Azospirillaceae | 1 | 1 | 1 | 0(1) | 0(0) | 0(0) | 0(0) | 0(0) | 0(0) |  |  |
| Cellvibrionaceae | 13 | 3 | 1 | 0(0) | 0(1) | 0(0) | 0(0) | 0(0) | 0(0) |  |  |
| Clostridiaceae_1 | 17 | 5 | 1 | 0(0) | 0(1) | 0(0) | 0(0) | 0(0) | 0(0) |  |  |
| Cytophagaceae | 32 | 7 | 1 | 0(0) | 0(1) | 0(0) | 0(0) | 0(0) | 0(0) |  |  |
| Demequinaceae | 2 | 1 | 1 | 0(0) | 0(1) | 0(0) | 0(0) | 0(0) | 0(0) |  |  |
| Frankiaceae | 4 | 3 | 1 | 0(0) | 0(0) | 0(0) | 0(0) | 0(1) | 0(0) |  |  |
| Geodermatophilaceae | 4 | 2 | 1 | 1(1) | 0(0) | 0(0) | 0(0) | 0(0) | 0(0) |  |  |
| Inquilinaceae | 6 | 5 | 1 | 0(0) | 0(0) | 0(0) | 0(0) | 0(0) | 1(1) |  |  |
| Intrasporangiaceae | 4 | 4 | 1 | 0(0) | 1(1) | 0(0) | 0(0) | 0(0) | 0(0) |  |  |
| JG30-KF-AS9 | 39 | 17 | 1 | 0(0) | 0(0) | 1(1) | 0(0) | 0(0) | 0(0) |  |  |
| KF-JG30-B3 | 6 | 5 | 1 | 0(0) | 0(0) | 0(0) | 0(0) | 0(0) | 0(1) |  |  |
| Longimicrobiaceae | 33 | 4 | 1 | 0(1) | 0(0) | 0(0) | 0(0) | 0(0) | 0(0) |  |  |
| Methyloligellaceae | 3 | 2 | 1 | 1(1) | 0(0) | 0(0) | 0(0) | 0(0) | 0(0) |  |  |
| Micrococcaceae | 4 | 1 | 1 | 0(0) | 1(1) | 0(0) | 0(0) | 0(0) | 0(0) |  |  |
| Micromonosporaceae | 30 | 16 | 1 | 0(0) | 0(0) | 0(0) | 0(0) | 0(1) | 0(0) |  |  |
| mle1-27 | 68 | 11 | 1 | 0(0) | 0(1) | 0(0) | 0(0) | 0(0) | 0(0) |  |  |
| Mycobacteriaceae | 11 | 10 | 1 | 0(0) | 1(1) | 0(0) | 0(0) | 0(0) | 0(0) |  |  |
| Nannocystaceae | 14 | 7 | 1 | 1(1) | 0(0) | 0(0) | 0(0) | 0(0) | 0(0) |  |  |
| Nitrospiraceae | 24 | 20 | 1 | 0(0) | 1(1) | 0(0) | 0(0) | 0(0) | 0(0) |  |  |
| NS9_marine_group | 17 | 4 | 1 | 0(0) | 0(1) | 0(0) | 0(0) | 0(0) | 0(0) |  |  |
| P3OB-42 | 57 | 10 | 1 | 0(0) | 0(1) | 0(0) | 0(0) | 0(0) | 0(0) |  |  |
| Peptostreptococcaceae | 4 | 3 | 1 | 0(0) | 0(1) | 0(0) | 0(0) | 0(0) | 0(0) |  |  |
| Puniceicoccaceae | 17 | 7 | 1 | 0(0) | 0(0) | 0(0) | 0(0) | 0(0) | 0(1) |  |  |
| Rhodobacteraceae | 15 | 8 | 1 | 0(0) | 0(1) | 0(0) | 0(0) | 0(0) | 0(0) |  |  |
| Rhodocyclaceae | 31 | 17 | 1 | 0(0) | 1(1) | 0(0) | 0(0) | 0(0) | 0(0) |  |  |
| Rhodomicrobiaceae | 2 | 2 | 1 | 0(0) | 0(0) | 0(0) | 0(0) | 0(1) | 0(0) |  |  |
| Rhodopirillaceae | 9 | 5 | 1 | 0(0) | 0(0) | 0(1) | 0(0) | 0(0) | 0(0) |  |  |
| Roseiflexaceae | 63 | 34 | 1 | 0(0) | 1(1) | 0(0) | 0(0) | 0(0) | 0(0) |  |  |
| Rubinisphaeraceae | 19 | 11 | 1 | 1(1) | 0(0) | 0(0) | 0(0) | 0(0) | 0(0) |  |  |
| Rubritaleaceae | 15 | 13 | 1 | 0(0) | 1(1) | 0(0) | 0(0) | 0(0) | 0(0) |  |  |
| SM2D12 | 97 | 15 | 1 | 0(0) | 0(0) | 0(0) | 0(0) | 0(0) | 0(1) |  |  |
| Tepidisphaeraceae | 13 | 8 | 1 | 0(1) | 0(0) | 0(0) | 0(0) | 0(0) | 0(0) |  |  |
| Thermoanaerobaculaceae | 70 | 28 | 1 | 0(1) | 0(0) | 0(0) | 0(0) | 0(0) | 0(0) |  |  |
| Saprospiraceae | 37 | 14 | 0 | 0(0) | 0(0) | 0(0) | 0(0) | 0(0) | 0(0) | VI | A |
| Sandaracinaceae | 90 | 20 | 0 | 0(0) | 0(0) | 0(0) | 0(0) | 0(0) | 0(0) | V | AG |
| Rhizobiaceae | 8 | 7 | 0 | 0(0) | 0(0) | 0(0) | 0(0) | 0(0) | 0(0) | I | AGF |
| unclassified  or incertae sedis | 9352 | 3068 | 208 | 29 (41) | 27 (84) | 9 (12) | 0 (0) | 20 (54) | 10 (17) |  |  |
| rest | 2211 | 431 | 0 | 0 | 0 | 0 | 0 | 0 | 0 |  |  |
| all | 18140 | 6979 | 699 | 86 (128) | 105 (287) | 23 (29) | 0 (4) | 42 (162) | 48 (89) |  |  |

^1)^ indicative and core OTUs: OTUs, which are indicative and core of the same land-use type(s), note that OTUs cannot be indicative of all sites, i.e., the combination AGF.

Table S8: Number of land-use-indicative fungal OTUs grouped by family. All families containing at least one indicative OTU, as well as families represented in Figure 4d are shown.

|  | Number of OTUs | | | indicative and core OTUs (all indicative OTUs)^1)^ | | | | | | Figure 4d | |
| --- | --- | --- | --- | --- | --- | --- | --- | --- | --- | --- | --- |
| Family | Total | site-core | indicative | A | AG | G | AF | FG | F | Cluster | Max. Abund |
| Lasiosphaeriaceae | 58 | 41 | 12 | 3(4) | 2(7) | 0(1) | 0(0) | 0(0) | 0(0) | III | A |
| Nectriaceae | 52 | 27 | 11 | 1(1) | 3(9) | 0(0) | 0(0) | 0(1) | 0(0) | III | A |
| Herpotrichiellaceae | 143 | 67 | 7 | 0(2) | 0(1) | 0(0) | 0(0) | 0(0) | 1(4) | IV | G |
| Mortierellaceae | 129 | 58 | 7 | 0(0) | 2(5) | 1(1) | 0(0) | 0(0) | 1(1) | IV | G |
| Helotiaceae | 120 | 44 | 7 | 0(2) | 3(4) | 0(0) | 0(0) | 0(0) | 0(1) | III | A |
| Hypocreaceae | 25 | 11 | 6 | 0(2) | 0(2) | 0(0) | 0(2) | 0(0) | 0(0) |  |  |
| Myxotrichaceae | 40 | 18 | 4 | 0(0) | 0(0) | 0(0) | 0(0) | 0(0) | 2(4) | V | F |
| Plectosphaerellaceae | 23 | 8 | 4 | 2(3) | 1(1) | 0(0) | 0(0) | 0(0) | 0(0) | III | A |
| Hyaloscyphaceae | 85 | 40 | 4 | 0(0) | 0(2) | 0(1) | 0(0) | 0(0) | 0(1) |  |  |
| Cucurbitariaceae | 14 | 9 | 3 | 0(0) | 0(2) | 1(1) | 0(0) | 0(0) | 0(0) | IV | G |
| Bulleribasidiaceae | 26 | 7 | 3 | 0(1) | 1(2) | 0(0) | 0(0) | 0(0) | 0(0) | III | AG |
| Chaetomiaceae | 39 | 20 | 3 | 2(2) | 0(0) | 0(1) | 0(0) | 0(0) | 0(0) | III | A |
| Mrakiaceae | 7 | 4 | 3 | 0(3) | 0(0) | 0(0) | 0(0) | 0(0) | 0(0) | III | A |
| Dermateaceae | 25 | 10 | 2 | 0(0) | 1(1) | 0(0) | 0(0) | 0(0) | 0(1) | IV | G |
| Tricholomataceae | 103 | 37 | 2 | 0(0) | 0(0) | 0(1) | 0(0) | 0(0) | 1(1) | IV | G |
| Clavicipitaceae | 35 | 19 | 2 | 0(0) | 0(2) | 0(0) | 0(0) | 0(0) | 0(0) | II | AG |
| Pseudeurotiaceae | 15 | 11 | 2 | 0(0) | 1(1) | 0(0) | 0(0) | 0(0) | 1(1) | II | AG |
| Chaetosphaeriaceae | 36 | 16 | 2 | 0(0) | 0(1) | 0(1) | 0(0) | 0(0) | 0(0) |  |  |
| Gloniaceae | 11 | 10 | 2 | 0(0) | 0(0) | 0(0) | 0(0) | 0(0) | 1(2) |  |  |
| Leucosporidiaceae | 7 | 3 | 2 | 0(0) | 0(2) | 0(0) | 0(0) | 0(0) | 0(0) |  |  |
| Phaeosphaeriaceae | 53 | 13 | 2 | 1(1) | 1(1) | 0(0) | 0(0) | 0(0) | 0(0) |  |  |
| Leotiaceae | 43 | 24 | 1 | 0(0) | 0(0) | 0(1) | 0(0) | 0(0) | 0(0) | V | F |
| Chaetothyriaceae | 4 | 2 | 1 | 0(0) | 0(0) | 0(1) | 0(0) | 0(0) | 0(0) | IV | G |
| Piskurozymaceae | 11 | 3 | 1 | 0(0) | 0(1) | 0(0) | 0(0) | 0(0) | 0(0) | III | A |
| Pyronemataceae | 92 | 38 | 1 | 0(0) | 0(1) | 0(0) | 0(0) | 0(0) | 0(0) | III | A |
| Bionectriaceae | 9 | 7 | 1 | 0(0) | 1(1) | 0(0) | 0(0) | 0(0) | 0(0) | II | AG |
| Myrmecridiaceae | 8 | 2 | 1 | 0(0) | 1(1) | 0(0) | 0(0) | 0(0) | 0(0) | II | AG |
| Sordariaceae | 4 | 1 | 1 | 0(0) | 1(1) | 0(0) | 0(0) | 0(0) | 0(0) | II | AG |
| Sporormiaceae | 23 | 5 | 1 | 0(0) | 1(1) | 0(0) | 0(0) | 0(0) | 0(0) | II | AG |
| Ascobolaceae | 13 | 7 | 1 | 0(0) | 0(1) | 0(0) | 0(0) | 0(0) | 0(0) |  |  |
| Aspergillaceae | 51 | 22 | 1 | 0(0) | 0(0) | 0(0) | 0(0) | 0(0) | 0(1) |  |  |
| Cystofilobasidiaceae | 5 | 2 | 1 | 0(1) | 0(0) | 0(0) | 0(0) | 0(0) | 0(0) | III | A |
| Didymellaceae | 7 | 2 | 1 | 0(0) | 1(1) | 0(0) | 0(0) | 0(0) | 0(0) |  |  |
| Didymosphaeriaceae | 6 | 3 | 1 | 0(0) | 0(1) | 0(0) | 0(0) | 0(0) | 0(0) |  |  |
| Filobasidiaceae | 10 | 1 | 1 | 0(1) | 0(0) | 0(0) | 0(0) | 0(0) | 0(0) |  |  |
| Hyponectriaceae | 4 | 2 | 1 | 0(0) | 0(1) | 0(0) | 0(0) | 0(0) | 0(0) |  |  |
| Leptosphaeriaceae | 19 | 5 | 1 | 0(1) | 0(0) | 0(0) | 0(0) | 0(0) | 0(0) |  |  |
| Magnaporthaceae | 17 | 5 | 1 | 0(0) | 0(1) | 0(0) | 0(0) | 0(0) | 0(0) |  |  |
| Melanommataceae | 5 | 2 | 1 | 0(0) | 0(1) | 0(0) | 0(0) | 0(0) | 0(0) |  |  |
| Microdochiaceae | 5 | 3 | 1 | 0(1) | 0(0) | 0(0) | 0(0) | 0(0) | 0(0) |  |  |
| Minutisphaeraceae | 6 | 2 | 1 | 0(0) | 0(0) | 1(1) | 0(0) | 0(0) | 0(0) |  |  |
| Mucoraceae | 21 | 9 | 1 | 0(0) | 0(1) | 0(0) | 0(0) | 0(0) | 0(0) |  |  |
| Periconiaceae | 6 | 2 | 1 | 1(1) | 0(0) | 0(0) | 0(0) | 0(0) | 0(0) |  |  |
| Pleosporaceae | 20 | 5 | 1 | 0(1) | 0(0) | 0(0) | 0(0) | 0(0) | 0(0) |  |  |
| Thyridariaceae | 2 | 1 | 1 | 0(0) | 0(1) | 0(0) | 0(0) | 0(0) | 0(0) |  |  |
| Torulaceae | 6 | 1 | 1 | 0(1) | 0(0) | 0(0) | 0(0) | 0(0) | 0(0) |  |  |
| Trichocomaceae | 18 | 9 | 1 | 0(1) | 0(0) | 0(0) | 0(0) | 0(0) | 0(0) |  |  |
| Trichosporonaceae | 7 | 3 | 1 | 0(0) | 1(1) | 0(0) | 0(0) | 0(0) | 0(0) |  |  |
| Venturiaceae | 38 | 22 | 1 | 0(0) | 0(0) | 0(0) | 0(0) | 0(0) | 1(1) |  |  |
| Vibrisseaceae | 10 | 5 | 1 | 0(0) | 0(0) | 0(0) | 0(0) | 0(0) | 0(1) |  |  |
| Atheliaceae | 42 | 26 | 0 | 0(0) | 0(0) | 0(0) | 0(0) | 0(0) | 0(0) | V | F |
| Clavulinaceae | 27 | 16 | 0 | 0(0) | 0(0) | 0(0) | 0(0) | 0(0) | 0(0) | V | F |
| Cortinariaceae | 129 | 48 | 0 | 0(0) | 0(0) | 0(0) | 0(0) | 0(0) | 0(0) | V | F |
| Elaphomycetaceae | 9 | 6 | 0 | 0(0) | 0(0) | 0(0) | 0(0) | 0(0) | 0(0) | V | F |
| Hydnodontaceae | 35 | 13 | 0 | 0(0) | 0(0) | 0(0) | 0(0) | 0(0) | 0(0) | V | F |
| Hygrophoraceae | 44 | 23 | 0 | 0(0) | 0(0) | 0(0) | 0(0) | 0(0) | 0(0) | V | F |
| Inocybaceae | 122 | 69 | 0 | 0(0) | 0(0) | 0(0) | 0(0) | 0(0) | 0(0) | V | F |
| Rhytismataceae | 20 | 6 | 0 | 0(0) | 0(0) | 0(0) | 0(0) | 0(0) | 0(0) | V | F |
| Russulaceae | 85 | 60 | 0 | 0(0) | 0(0) | 0(0) | 0(0) | 0(0) | 0(0) | V | F |
| Sebacinaceae | 64 | 43 | 0 | 0(0) | 0(0) | 0(0) | 0(0) | 0(0) | 0(0) | V | F |
| Thelephoraceae | 136 | 70 | 0 | 0(0) | 0(0) | 0(0) | 0(0) | 0(0) | 0(0) | V | F |
| Clavariaceae | 166 | 71 | 0 | 0(0) | 0(0) | 0(0) | 0(0) | 0(0) | 0(0) | IV | G |
| Ophiocordycipitaceae | 20 | 7 | 0 | 0(0) | 0(0) | 0(0) | 0(0) | 0(0) | 0(0) | IV | G |
| Trimorphomycetaceae | 5 | 2 | 0 | 0(0) | 0(0) | 0(0) | 0(0) | 0(0) | 0(0) | I | AGF |
| unclassified or incertae sedis | 4256 | 1187 | 52 | 6(18) | 4(21) | 3(6) | 0(0) | 0(0) | 3(7) |  |  |
| Rest | 1801 | 487 | 0 | 0 | 0 | 0 | 0 | 0 | 0 |  |  |
| All | 8477 | 2802 | 171 | 16(47) | 25(79) | 6(16) | 0(2) | 0(1) | 11(26) |  |  |

^1)^ indicative and core OTUs: OTUs, which are indicative and core of the same land-use type(s), note that OTUs cannot be indicative of all sites, i.e., the combination AGF.

Table S9: Relative abundances of bacterial phyla in arable land, permanent grassland, and forest. Mean values (± standard deviations) of each land-use type are shown. Different letters indicate significant differences (Dunn Test, p < 0.05).

| Phylum |  | Arable land (A)  [%] | | | Permanent  Grassland (G) [%] | | | | Forest (F)  [%] | | | |  | Pattern^1)^ |
| --- | --- | --- | --- | --- | --- | --- | --- | --- | --- | --- | --- | --- | --- | --- |
| Proteobacteria |  | 22.4 | ± | 1.97 |  | 21.1 | ± | 2.12 |  | 26.8 | ± | 4.21 |  | G<A<F |
| Verrucomicrobia |  | 14.2 | ± | 4.03 |  | 20.4 | ± | 5.22 |  | 22.7 | ± | 9.01 |  | A<G=F |
| Acidobacteria |  | 14.3 | ± | 2.05 |  | 16.1 | ± | 5.41 |  | 17.1 | ± | 6.78 |  | A=G=F |
| Planctomycetes |  | 9.3 | ± | 1.96 |  | 9.6 | ± | 1.49 |  | 7.3 | ± | 1.82 |  | A=G>F |
| Chloroflexi |  | 9.1 | ± | 2.00 |  | 7.3 | ± | 2.35 |  | 5.1 | ± | 2.92 |  | A>G>F |
| Bacteroidetes |  | 7.6 | ± | 2.17 |  | 5.6 | ± | 1.98 |  | 3.5 | ± | 1.29 |  | A>G>F |
| Actinobacteria |  | 4.8 | ± | 1.43 |  | 3.4 | ± | 1.19 |  | 3.7 | ± | 1.53 |  | A>G=F |
| Patescibacteria |  | 1.9 | ± | 1.15 |  | 1.9 | ± | 0.93 |  | 1.9 | ± | 1.14 |  | A=G=F |
| Gemmatimonadetes |  | 2.7 | ± | 0.59 |  | 1.5 | ± | 0.46 |  | 1.1 | ± | 0.60 |  | A>G>F |
| Latescibacteria |  | 1.4 | ± | 0.45 |  | 1.8 | ± | 0.84 |  | 0.4 | ± | 0.49 |  | A=G>F |
| Rokubacteria |  | 1.0 | ± | 0.37 |  | 1.3 | ± | 0.49 |  | 0.8 | ± | 0.80 |  | A=G>F |
| Nitrospirae |  | 0.8 | ± | 0.38 |  | 0.9 | ± | 0.53 |  | 0.4 | ± | 0.58 |  | A=G>F |
| Candidate WPS-2 |  | 0.0 | ± | 0.00 |  | 0.2 | ± | 0.48 |  | 0.8 | ± | 1.17 |  | A<G<F |
| Armatimonadetes |  | 0.4 | ± | 0.20 |  | 0.1 | ± | 0.14 |  | 0.3 | ± | 0.22 |  | A>F>G |
| Elusimicrobia |  | 0.2 | ± | 0.08 |  | 0.3 | ± | 0.08 |  | 0.2 | ± | 0.08 |  | A=F<G |
| Cyanobacteria |  | 0.1 | ± | 0.04 |  | 0.1 | ± | 0.17 |  | 0.3 | ± | 0.31 |  | A=G<F |
| Firmicutes |  | 0.2 | ± | 0.13 |  | 0.2 | ± | 0.12 |  | 0.0 | ± | 0.05 |  | A=G>F |
| Chlamydiae |  | 0.0 | ± | 0.02 |  | 0.0 | ± | 0.06 |  | 0.2 | ± | 0.20 |  | A<G<F |
| Fibrobacteres |  | 0.1 | ± | 0.04 |  | 0.1 | ± | 0.06 |  | 0.0 | ± | 0.03 |  | G>A>F |
| Candidate FCPU426 |  | 0.0 | ± | 0.03 |  | 0.1 | ± | 0.04 |  | 0.1 | ± | 0.11 |  | A<G=F |
| Hydrogenedentes |  | 0.1 | ± | 0.06 |  | 0.0 | ± | 0.01 |  | 0.0 | ± | 0.02 |  | A>G=F |
| Dependentiae |  | 0.0 | ± | 0.00 |  | 0.0 | ± | 0.02 |  | 0.0 | ± | 0.08 |  | A<G<F |
| Entotheonellaeota |  | 0.0 | ± | 0.02 |  | 0.0 | ± | 0.01 |  | 0.0 | ± | 0.09 |  | A>G=F |
| Candidate BRC1 |  | 0.0 | ± | 0.01 |  | 0.0 | ± | 0.01 |  | 0.0 | ± | 0.01 |  | A>G>F |
| Candidate WS2 |  | 0.0 | ± | 0.02 |  | 0.0 | ± | 0.01 |  | 0.0 | ± | 0.01 |  | A>G>F |
| Omnitrophicaeota |  | 0.0 | ± | 0.04 |  | 0.0 | ± | 0.01 |  | 0.0 | ± | 0.01 |  | A>G=F |
| Candidate GAL15 |  | 0.0 | ± | 0.01 |  | 0.0 | ± | 0.01 |  | 0.0 | ± | 0.04 |  | A=G=F |
| Zixibacteria |  | 0.0 | ± | 0.03 |  | 0.0 | ± | 0.01 |  | 0.0 | ± | 0.01 |  | A=G=F |
| Spirochaetes |  | 0.0 | ± | 0.00 |  | 0.0 | ± | 0.01 |  | 0.0 | ± | 0.03 |  | G>A=F |
| Dadabacteria |  | 0.0 | ± | 0.01 |  | 0.0 | ± | 0.00 |  | 0.0 | ± | 0.01 |  | A=G=F |
| Candidate WS4 |  | 0.0 | ± | 0.00 |  | 0.0 | ± | 0.00 |  | 0.0 | ± | 0.00 |  | A=G=F |
| Unclassified |  | 0.3 | ± | 0.14 |  | 0.3 | ± | 0.08 |  | 0.1 | ± | 0.07 |  | A=G>F |

^1)^ Pattern: < and > operators indicate significant differences in relative abundance between land-use types, A: arable land, G: grassland, F: forest.

Table S10 Relative abundances of fungal phyla in arable land, permanent grassland, and forest. Mean values (± standard deviations) of each land-use type are shown. Different letters indicate significant differences (Dunn Test, p < 0.05).

| Phylum |  | Arable land (A) | | |  | Permanent  Grassland (G) | | |  | Forest (F) | | |  | Pattern^1)^ |
| --- | --- | --- | --- | --- | --- | --- | --- | --- | --- | --- | --- | --- | --- | --- |
| Ascomycota |  | 55.4 | ± | 7.80 |  | 53.2 | ± | 12.56 |  | 22.2 | ± | 9.97 |  | A=G>F |
| Basidiomycota |  | 11.7 | ± | 5.43 |  | 20.3 | ± | 18.55 |  | 55.7 | ± | 12.90 |  | A=G<F |
| Mortierellomycota |  | 3.4 | ± | 2.42 |  | 7.3 | ± | 6.55 |  | 1.2 | ± | 1.02 |  | G>A>F |
| Chytridiomycota |  | 2.1 | ± | 2.24 |  | 0.6 | ± | 0.61 |  | 0.0 | ± | 0.01 |  | A>G>F |
| Glomeromycota |  | 1.1 | ± | 1.25 |  | 1.3 | ± | 1.07 |  | 0.0 | ± | 0.03 |  | G=A>F |
| Mucoromycota |  | 0.1 | ± | 0.16 |  | 0.1 | ± | 0.08 |  | 0.5 | ± | 0.67 |  | A=G=F |
| Rozellomycota |  | 0.0 | ± | 0.17 |  | 0.1 | ± | 0.10 |  | 0.0 | ± | 0.05 |  | A<F<G |
| Olpidiomycota |  | 0.0 | ± | 0.05 |  | 0.0 | ± | 0.04 |  | 0.0 | ± | 0.00 |  | A=G>F |
| Blastocladiomycota |  | 0.0 | ± | 0.01 |  | 0.0 | ± | 0.02 |  | 0.0 | ± | 0.00 |  | G>A=F |
| unclassified |  | 1.7 | ± | 1.41 |  | 1.0 | ± | 0.62 |  | 0.3 | ± | 0.37 |  | A>G>F |

^1)^ Pattern: < and > operators indicate significant differences in relative abundance between land-use types, A: arable land, G: grassland, F: forest.
